# Supplementary figures and images for: Genomic anatomy of male-specific microchromosomes in a gynogenetic fish
Source: PLoS Genet. 2021 Sep 7;17(9):e1009760. doi: 10.1371/journal.pgen.1009760 (PMC8448357; doi:10.1371/journal.pgen.1009760)

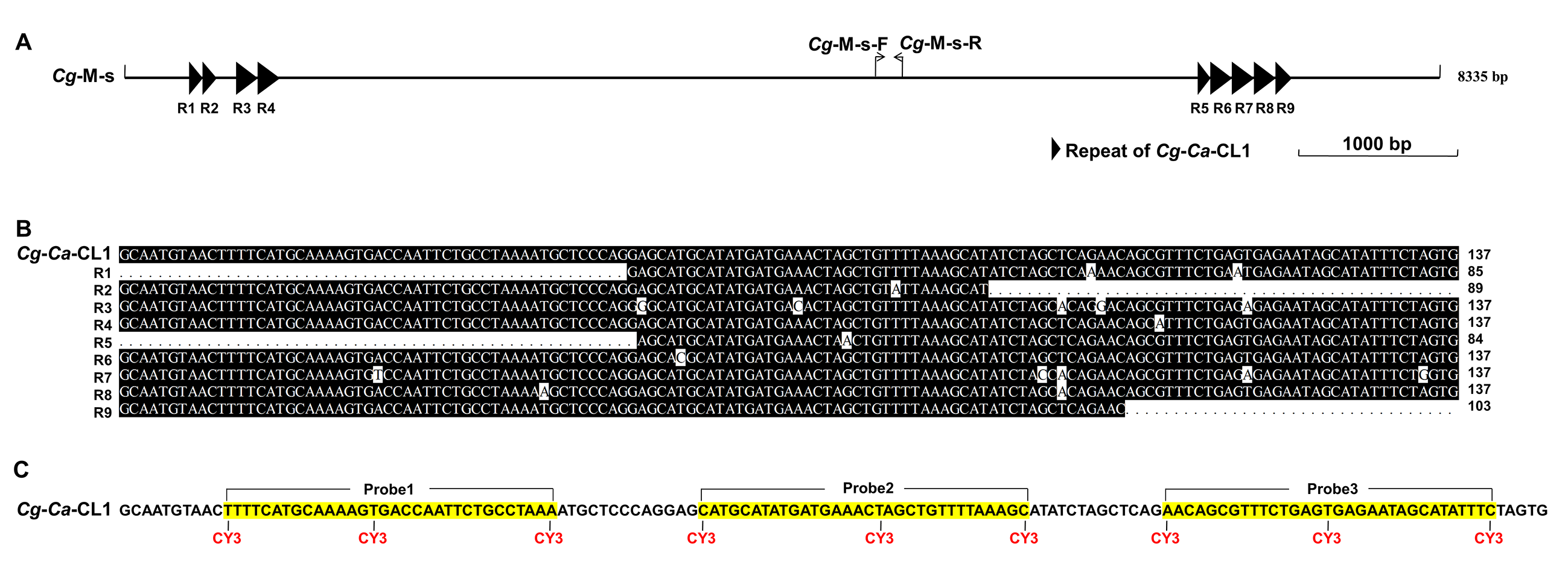

Supplement: S1 Fig — (A) Cg-M-s contains several intact and fragmental repeats of Cg-Ca-CL1. The sites of male-specific primers including Cg-M-s-F and Cg-M-s-R are marked by black arrows. (B) Sequence alignment between the consensus sequence of Cg-Ca-CL1 and the repeats of Cg-Ca-CL1 in Cg-M-s. (C) Peptide nucleic acid (PNA) probes used for fluorescence in situ hybridization (FISH). The sequences of PNA probes are indicated by yellow background. Each probe is labeled with three Cy3. (TIF) [file pgen.1009760.s001.tif]

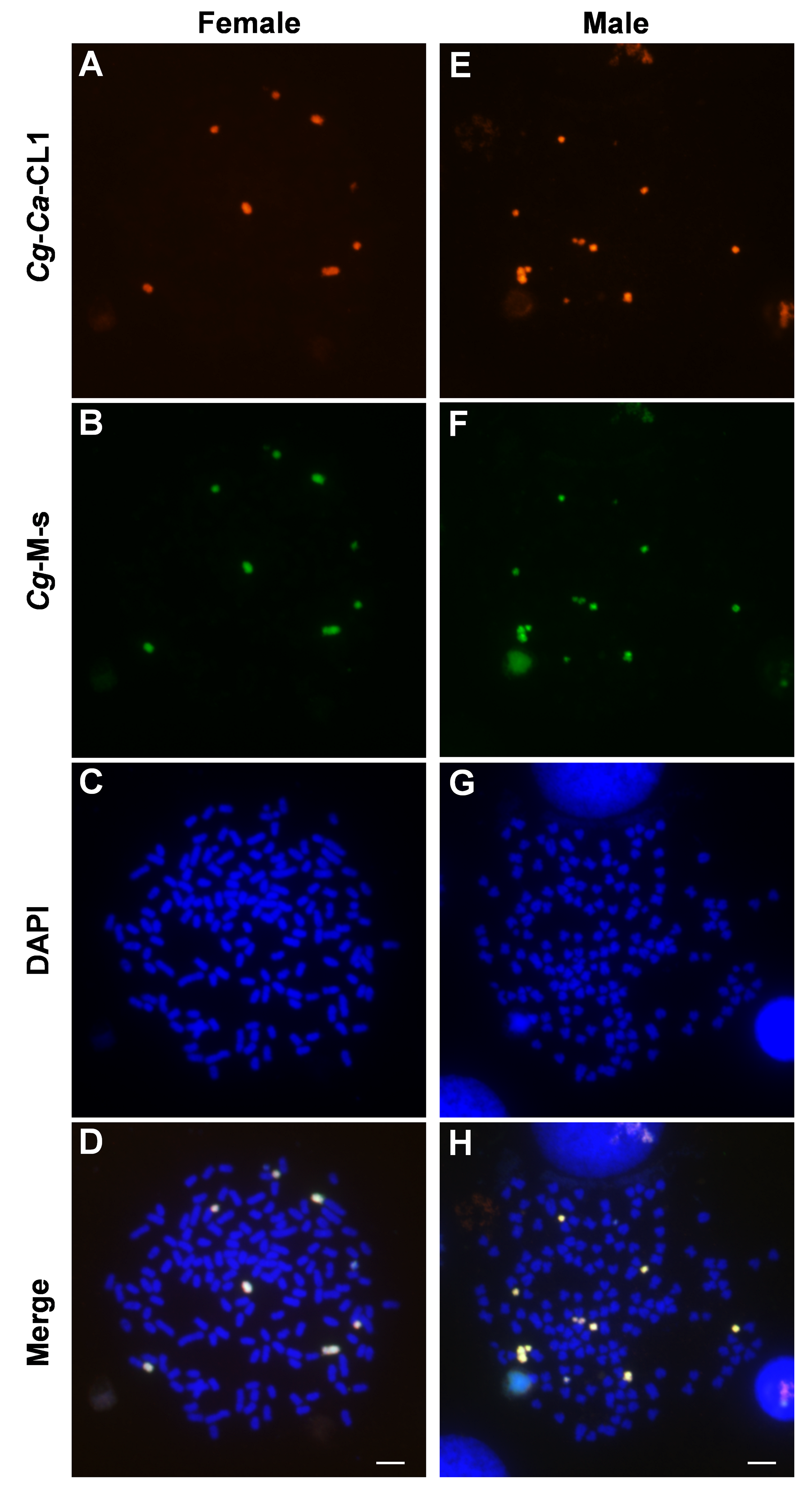

Supplement: S2 Fig — (A-H) The Cg-Ca-CL1 probe and Cg-M-s probe were labeled with Biotin and Digoxin respectively, and red and green fluorescence were produced accordingly. FISH analysis was performed in metaphases of female C. gibelio (A-D) and male C. gibelio (E-H). Chromosomes were counterstained with DAPI and appeared blue. Scale bar = 5 μm. (TIF) [file pgen.1009760.s002.tif]

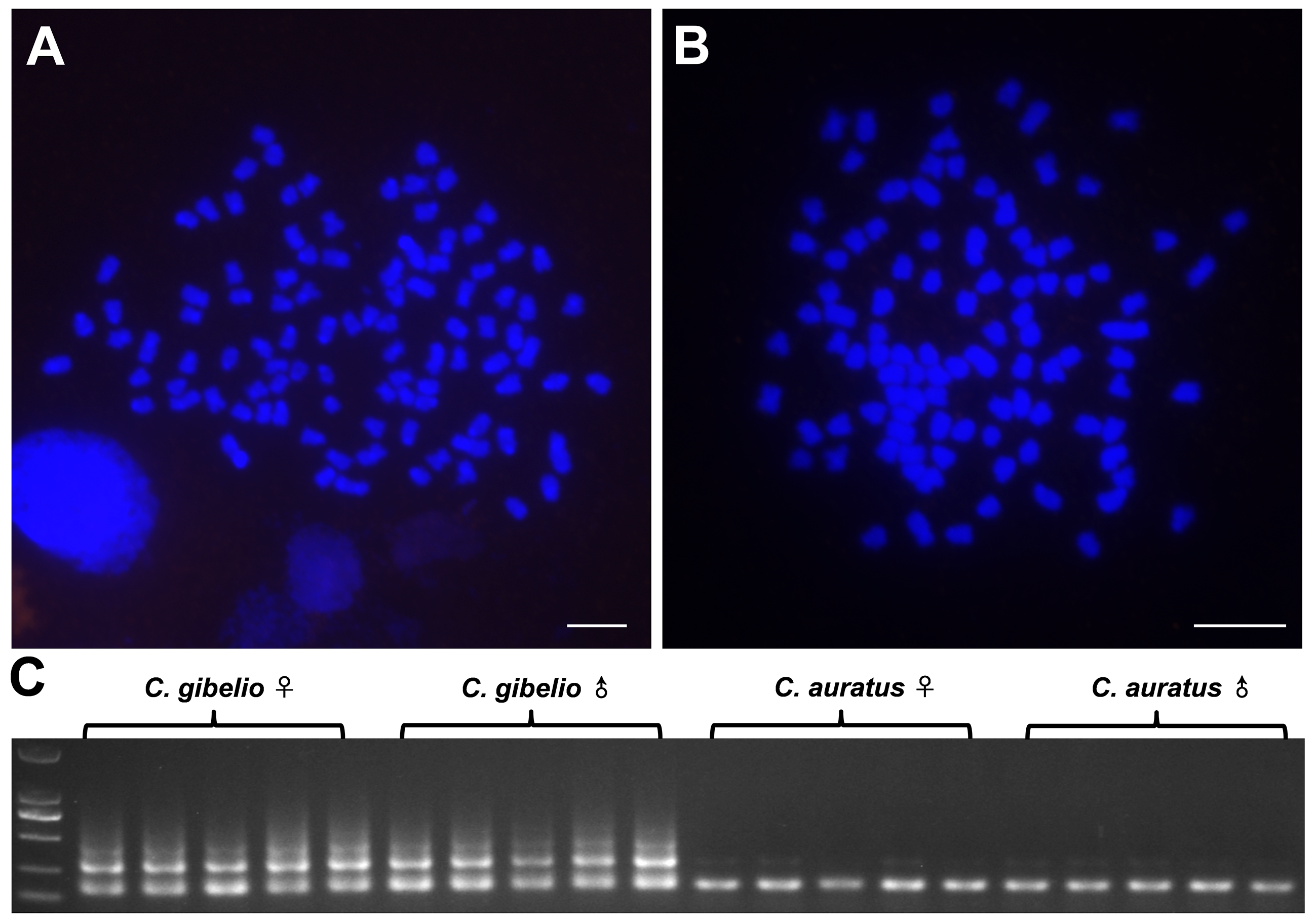

Supplement: S3 Fig — (A, B) FISH analysis of satellite repeat cluster Cg-Ca-CL1 (red) in metaphases of female C. auratus (A) and male C. auratus (B). (C) PCR assay of satellite repeat cluster Cg-Ca-CL1 in C. gibelio and C. Carassius. ♀, female; ♂, male. Scale bar = 5 μm. (TIF) [file pgen.1009760.s003.tif]

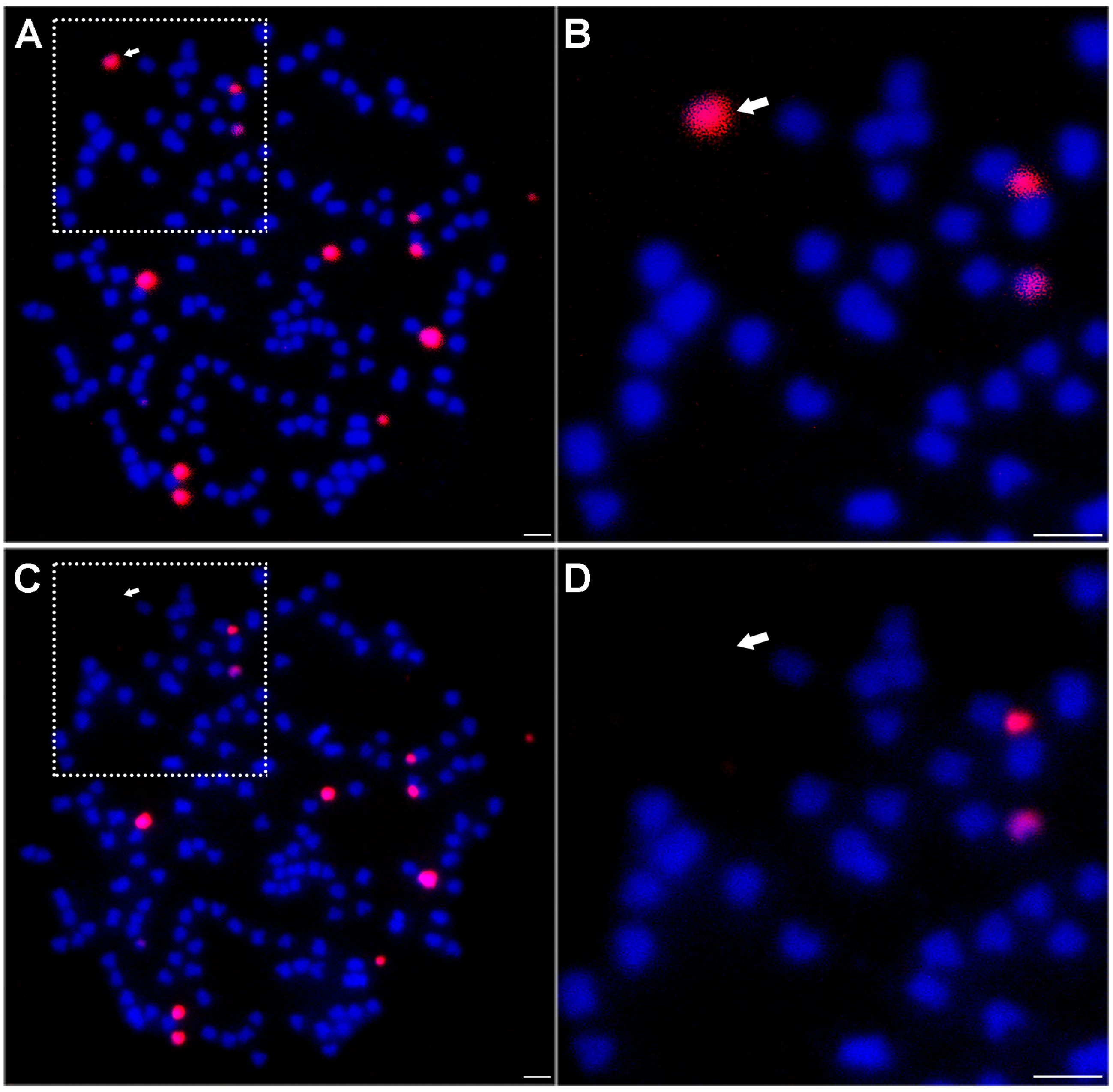

Supplement: S4 Fig — (A, C) FISH detection on a male metaphase using PNA probes before (A) and after (C) microdissection. (B, D) The enlarged images of the white squares in (A) and (C), respectively. The red signals from PNA probes highlighted microchromosomes, and chromosomes were counterstained with DAPI and appeared blue. The white arrows indicate chromosome before (A and B) and after (C and D) isolation. Scale bar = 5 μm. (TIF) [file pgen.1009760.s004.tif]

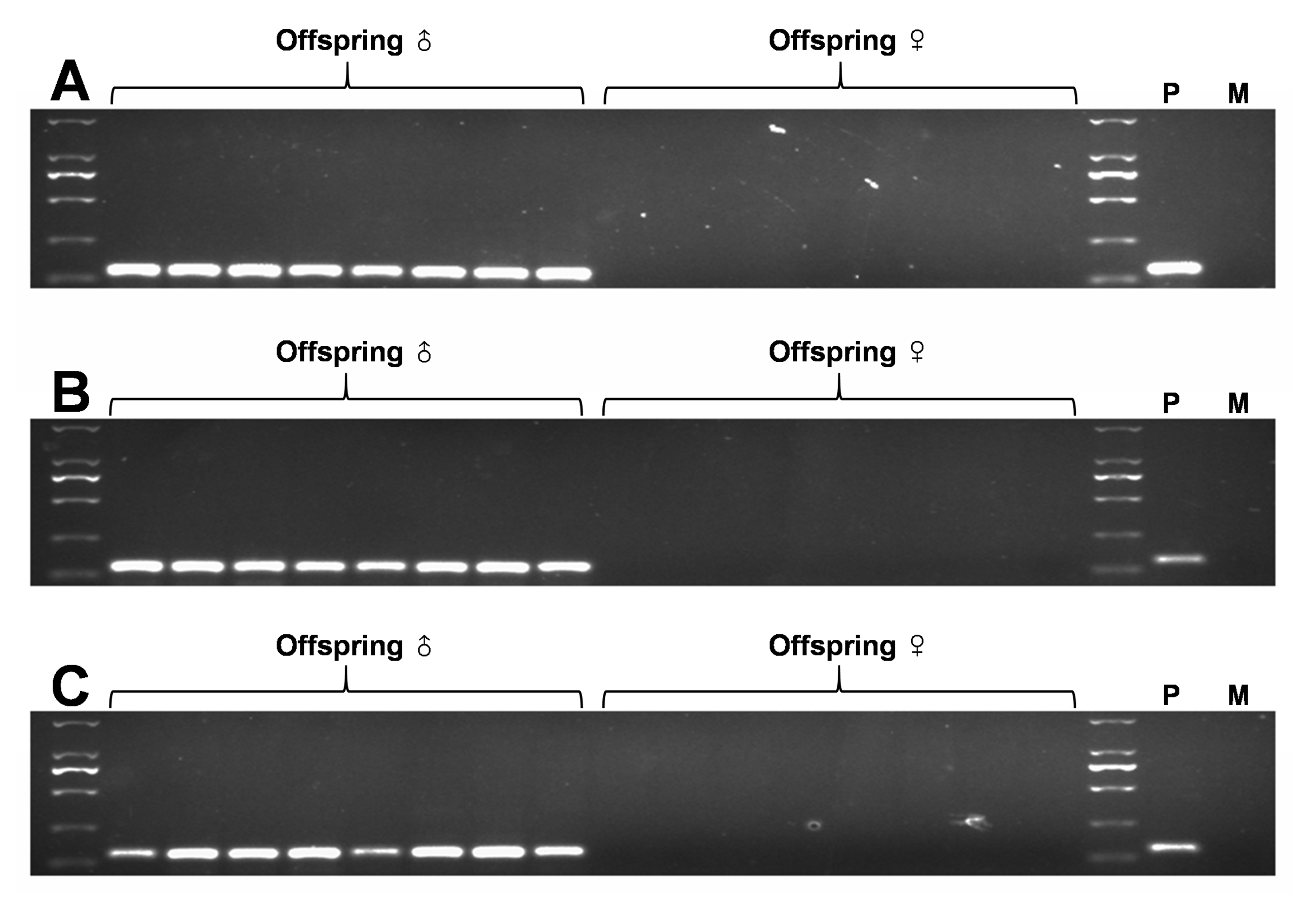

Supplement: S5 Fig — (A-C) Male-specific marker in 8 randomly-picked males and 8 randomly-picked females in the offspring as well as the parental individuals from family 1 (A), family 2 (B), and family 3 (C). ♀, female; ♂, male; M, maternal individual; P, paternal individual. (TIF) [file pgen.1009760.s005.tif]

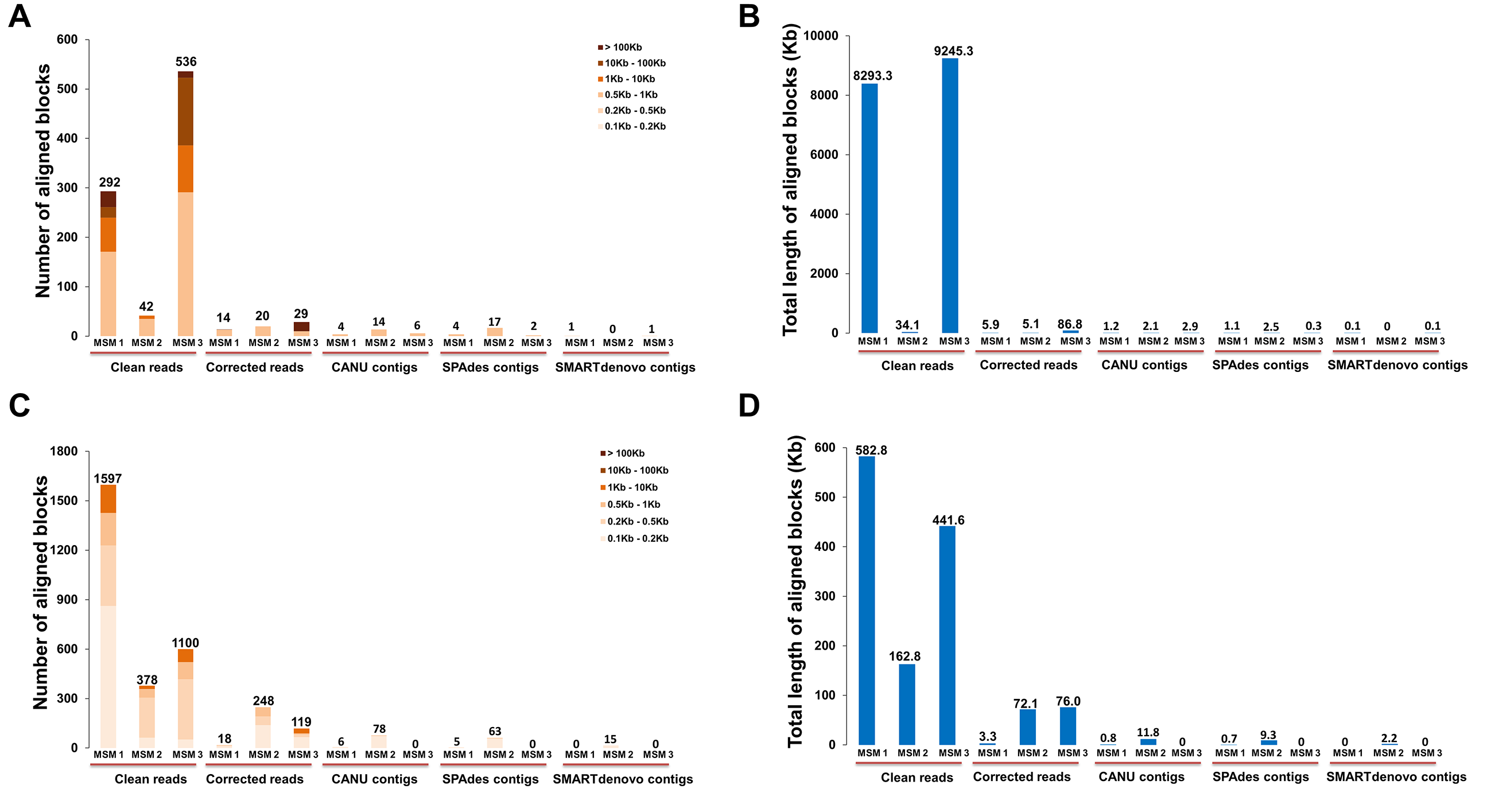

Supplement: S6 Fig — (A, B) Number (A) and total length (B) of aligned blocks referring to the female genome. (C, D) Number (C) and total length (D) of aligned blocks referring to the full-length transcriptomes. Different colors represent different lengths of aligned blocks. The X axis represents different datasets including clean reads, corrected reads, contigs assembled by CANU, contigs assembled by SPAdes, and contigs assemble by SMARTdenovo. (TIF) [file pgen.1009760.s006.tif]

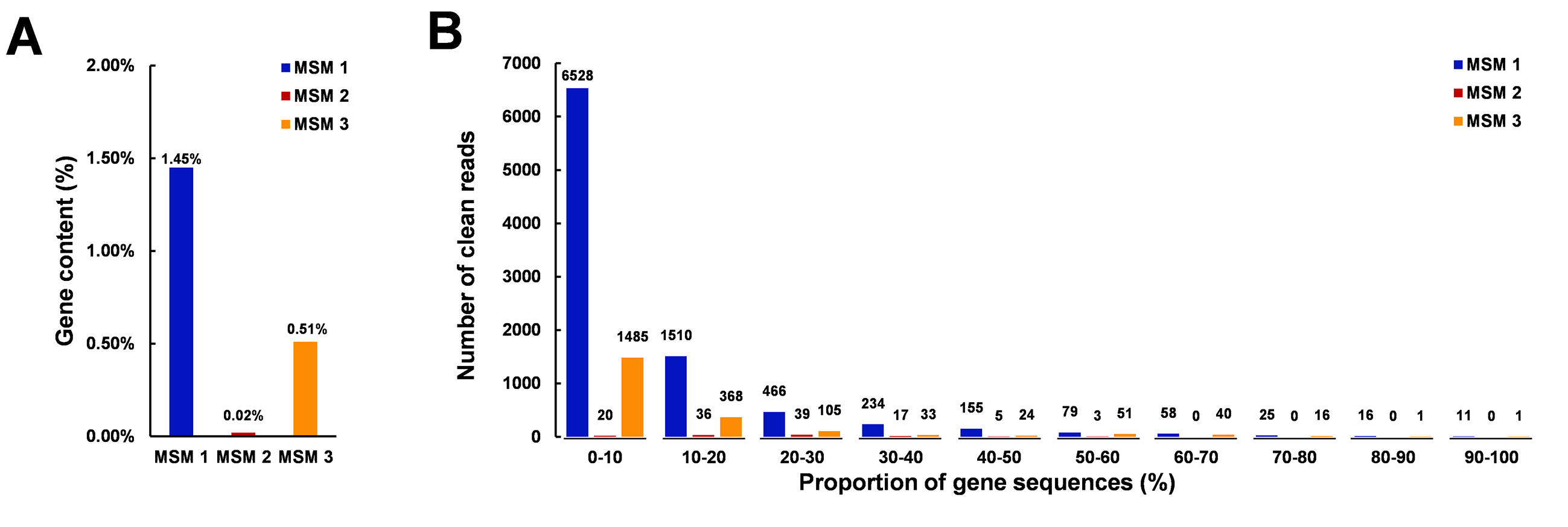

Supplement: S7 Fig — (A) Gene content of three MSMs. (B) Number of clean reads containing gene sequences. The X axis represents the proportion of gene sequences. The Y axis indicates the number of clean reads. (TIF) [file pgen.1009760.s007.tif]

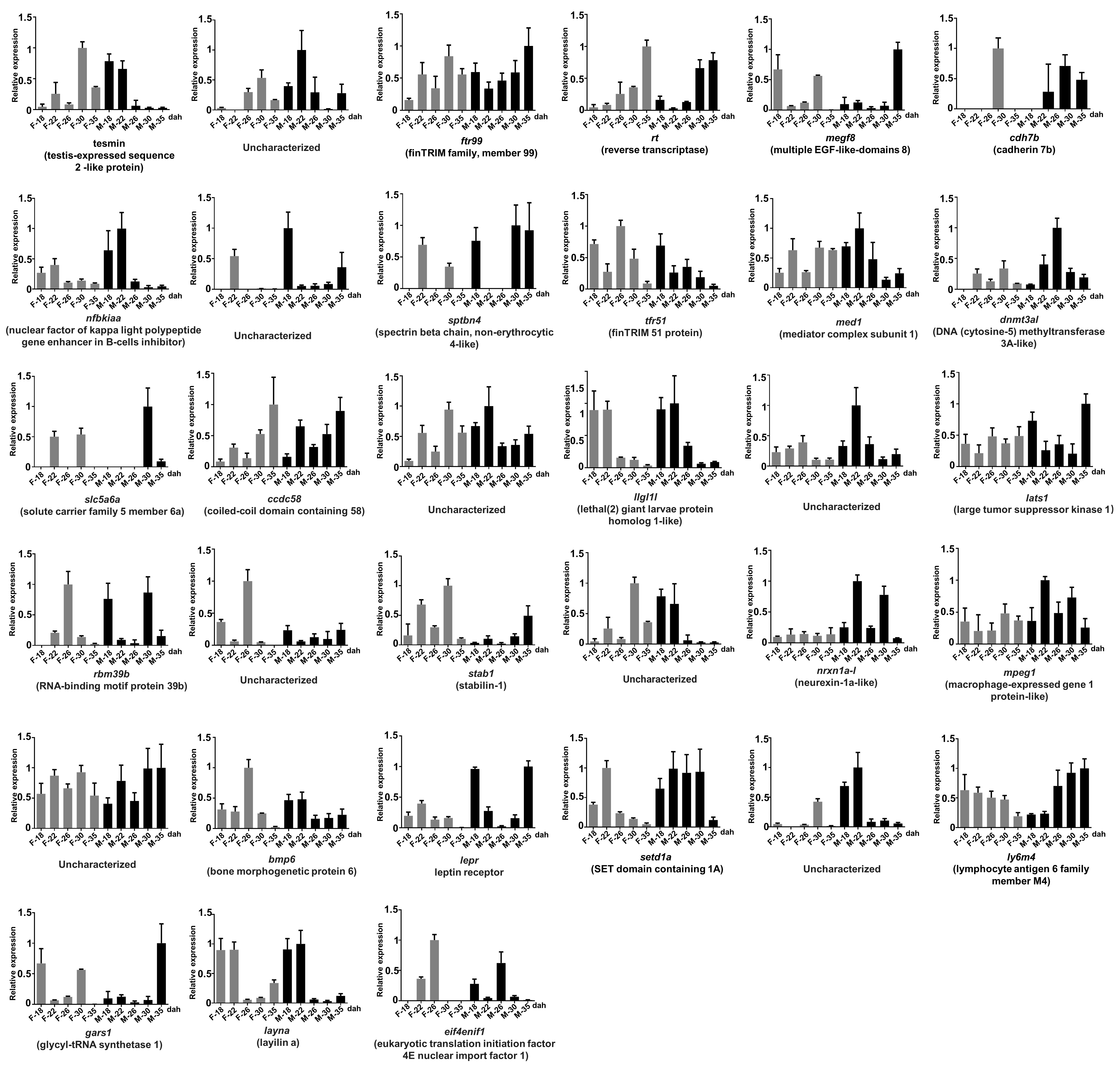

Supplement: S8 Fig — qPCR detection of gene fragments at early gonadal developmental stages including 18, 22, 26, 30, and 35 dah (days after hatch). The X axis represents the stages of gonad development. The Y axis represents the relative expression, and the highest expression level of each gene fragment was used as control and defined as 1. F, female; M, male. (TIF) [file pgen.1009760.s008.tif]

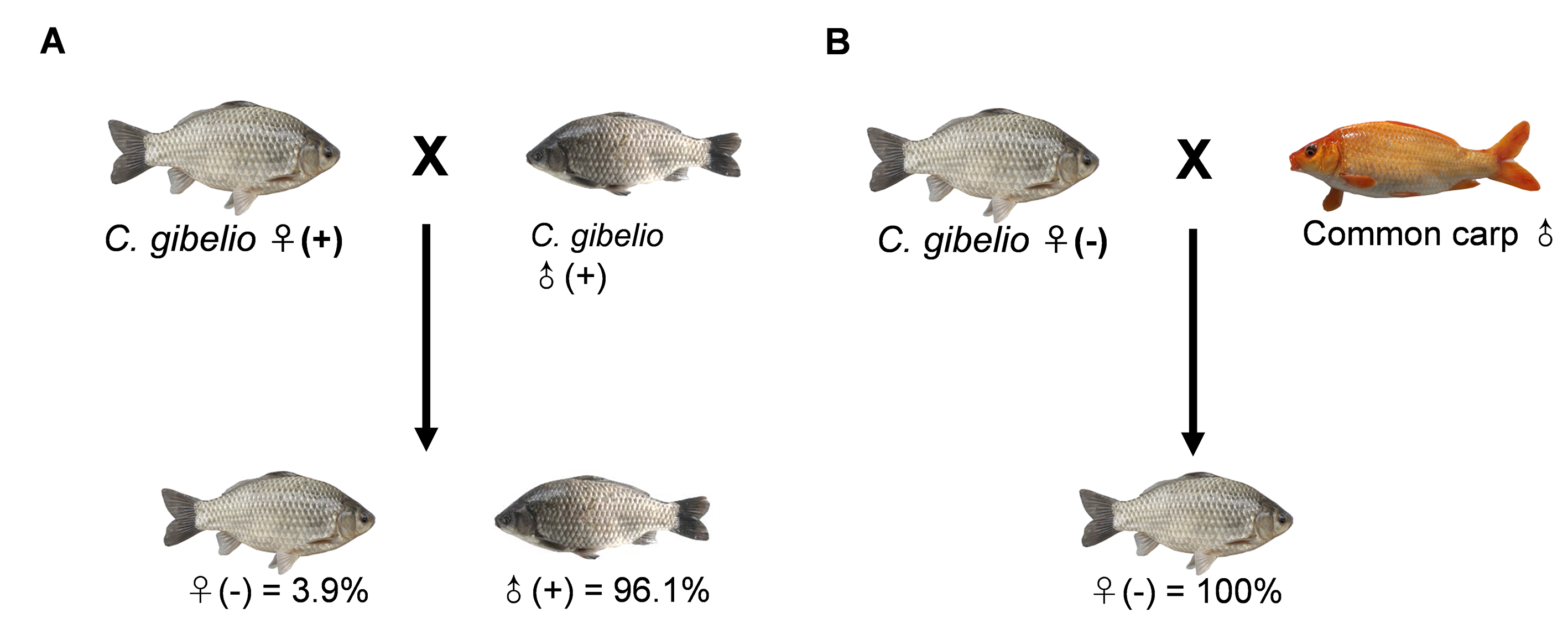

Supplement: S9 Fig — (A) Establishment of a family containing a high proportion of male offspring. (B) Establishment of a family with all-female offspring. ♀, female; ♂, male; (+), with the male-specific genetic marker; (-) without the male-specific genetic marker. (TIF) [file pgen.1009760.s009.tif]
