# Supplementary material for: Genomic anatomy of male-specific microchromosomes in a gynogenetic fish
Source: PLoS Genet. 2021 Sep 7;17(9):e1009760. doi: 10.1371/journal.pgen.1009760 (PMC8448357; doi:10.1371/journal.pgen.1009760)
Supplement: S1 Table — (DOCX) [file pgen.1009760.s010.docx]

**Supplementary Table 1 - The consensus sequence of satellite DNA *Cg*-*Ca*-CL1.**

| **Name** | **Sequence (5'-3')** |
| --- | --- |
| *Cg*-*Ca*-CL1 | GCAATGTAACTTTTCATGCAAAAGTGACCAATTCTGCCTAAAATGCTCCCAGGAGCATGCATATGATGAAACTAGCTGTTTTAAAGCATATCTAGCTCAGAACAGCGTTTCTGAGTGAGAATAGCATATTTCTAGTG |
