# Supplementary material for: Genomic anatomy of male-specific microchromosomes in a gynogenetic fish
Source: PLoS Genet. 2021 Sep 7;17(9):e1009760. doi: 10.1371/journal.pgen.1009760 (PMC8448357; doi:10.1371/journal.pgen.1009760)
Supplement: S2 Table — (DOCX) [file pgen.1009760.s011.docx]

**Supplementary Table 2 -** **Sequencing summary of male-specific microchromosomes.**

| **Sample** | **Technical Platform** | **Number of reads** | **Data size (Gb)** | **Read N50 (bp)** | **Longest read (bp)** | **Average length**  **(bp)** | **Number of genes** |
| --- | --- | --- | --- | --- | --- | --- | --- |
| Male-specific microchromosome 1 | PacBio | 624,541 | 2.56 | 7,429 | 167,138 | 4,259 | 228 |
| Male-specific microchromosome 2 | PacBio | 505,551 | 2.34 | 8,127 | 146,177 | 4,457 | 88 |
| Male-specific microchromosome 3 | PacBio | 415,341 | 2.02 | 8,367 | 148,530 | 4,686 | 203 |
| Male-specific microchromosomes | - | 1,545,433 | 6.92 | 7,974 | 167,138 | 4,467 | 487 |
