# Supplementary material for: Genomic anatomy of male-specific microchromosomes in a gynogenetic fish
Source: PLoS Genet. 2021 Sep 7;17(9):e1009760. doi: 10.1371/journal.pgen.1009760 (PMC8448357; doi:10.1371/journal.pgen.1009760)
Supplement: S3 Table — (DOCX) [file pgen.1009760.s012.docx]

**Supplementary Table 3** **- The summary of the corrected sequences of MSMs.**

|  | Corrected reads of MSM 1 | | Corrected reads of MSM 2 | | Corrected reads of MSM 3 | |
| --- | --- | --- | --- | --- | --- | --- |
|  | Length (bp) | Number | Length (bp) | Number | Length (bp) | Number |
| N90 | 1,380 | 134,562 | 1,556 | 158,774 | 1,608 | 156,975 |
| N80 | 2,086 | 99,781 | 2,274 | 119,790 | 2,343 | 119,293 |
| N70 | 2,803 | 75,239 | 2,976 | 91,418 | 3,048 | 91,738 |
| N60 | 3,587 | 56,482 | 3,733 | 69,246 | 3,761 | 69,985 |
| N50 | 4,452 | 41,595 | 4,531 | 51,279 | 4,495 | 52,028 |
| N40 | 5,461 | 29,510 | 5,496 | 36,415 | 5,359 | 36,967 |
| N30 | 6,614 | 19,590 | 6,792 | 24,285 | 6,473 | 24,396 |
| N20 | 8,075 | 11,447 | 8,261 | 14,438 | 8,044 | 14,112 |
| N10 | 10,036 | 4,757 | 9,899 | 6,194 | 10,177 | 5,871 |
| Longest reads | 35,697 |  | 40,555 |  | 51,229 |  |
| Total_size | 594,989,677 |  | 739,457,098 |  | 738,267,061 |  |
| Corrected reads | >= 100 bp | 200,170 | >= 100 bp | 231,778 | >= 100 bp | 228,337 |
| Corrected reads | >= 2 kb | 103,331 | >= 2 kb | 133,378 | >= 2 kb | 135,703 |
| GC rate (%) | 40.3 | | 40.2 | | 39.1 | |
