# Supplementary material for: Genomic anatomy of male-specific microchromosomes in a gynogenetic fish
Source: PLoS Genet. 2021 Sep 7;17(9):e1009760. doi: 10.1371/journal.pgen.1009760 (PMC8448357; doi:10.1371/journal.pgen.1009760)
Supplement: S4 Table — (DOCX) [file pgen.1009760.s013.docx]

**Supplementary Table 4 -** **The summary of sequence assembly of MSMs by CANU.**

|  | Assembled contig of MSM 1 | | Assembled contig of MSM 2 | | Assembled contig of MSM 3 | |
| --- | --- | --- | --- | --- | --- | --- |
|  | Length (bp) | Number | Length (bp) | Number | Length (bp) | Number |
| N90 | 10,140 | 333 | 10,483 | 407 | 11,200 | 318 |
| N80 | 12,642 | 260 | 13,972 | 323 | 15,330 | 246 |
| N70 | 15,303 | 201 | 16,365 | 252 | 18,379 | 190 |
| N60 | 18,095 | 151 | 18,795 | 192 | 21,985 | 143 |
| N50 | 21,355 | 109 | 22,557 | 140 | 25,402 | 103 |
| N40 | 26,777 | 74 | 26,402 | 96 | 29,469 | 69 |
| N30 | 32,915 | 46 | 33,647 | 59 | 42,895 | 42 |
| N20 | 44,771 | 24 | 43,235 | 31 | 53,225 | 22 |
| N10 | 73,623 | 9 | 71,946 | 11 | 80,450 | 7 |
| Longest contigs | 170,906 |  | 204,227 |  | 277,547 |  |
| Total_size | 8,265,242 |  | 10,644,891 |  | 9,461,102 |  |
| Assembled contigs | >= 100 bp | 457 | >= 100 bp | 561 | >= 100 bp | 446 |
| Assembled contigs | >= 2 kb | 456 | >= 2 kb | 557 | >= 2 kb | 445 |
| GC rate (%) | 39.9 | | 40.6 | | 38.7 | |
