# Supplementary material for: Genomic anatomy of male-specific microchromosomes in a gynogenetic fish
Source: PLoS Genet. 2021 Sep 7;17(9):e1009760. doi: 10.1371/journal.pgen.1009760 (PMC8448357; doi:10.1371/journal.pgen.1009760)
Supplement: S5 Table — (DOCX) [file pgen.1009760.s014.docx]

**Supplementary Table 5 -** **The summary of sequence assembly of MSMs by SPAdes.**

|  | Assembled contig of MSM 1 | | Assembled contig of MSM 2 | | Assembled contig of MSM 3 | |
| --- | --- | --- | --- | --- | --- | --- |
|  | Length (bp) | Number | Length (bp) | Number | Length (bp) | Number |
| N90 | 201 | 428 | 174 | 1,270 | 188 | 553 |
| N80 | 376 | 287 | 230 | 828 | 250 | 305 |
| N70 | 567 | 199 | 336 | 473 | 737 | 181 |
| N60 | 776 | 139 | 678 | 291 | 1,187 | 123 |
| N50 | 1,089 | 95 | 1,084 | 184 | 1,570 | 83 |
| N40 | 1,482 | 63 | 1,609 | 115 | 2,196 | 54 |
| N30 | 1,814 | 38 | 2,351 | 68 | 3,075 | 32 |
| N20 | 2,654 | 19 | 3,289 | 35 | 4,382 | 17 |
| N10 | 4,051 | 6 | 5,026 | 13 | 6,357 | 7 |
| Longest contigs | 16,732 |  | 14,078 |  | 15,225 |  |
| Total_size | 402,155 |  | 901,525 |  | 549,918 |  |
| Assembled contigs | >= 100 bp | 759 | >= 100 bp | 1,878 | >= 100 bp | 917 |
| Assembled contigs | >= 2 kb | 29 | >= 2 kb | 82 | >= 2 kb | 64 |
| GC rate (%) | 40.0 | | 39.9 | | 39.0 | |
