# Supplementary material for: Genomic anatomy of male-specific microchromosomes in a gynogenetic fish
Source: PLoS Genet. 2021 Sep 7;17(9):e1009760. doi: 10.1371/journal.pgen.1009760 (PMC8448357; doi:10.1371/journal.pgen.1009760)
Supplement: S6 Table — (DOCX) [file pgen.1009760.s015.docx]

**Supplementary Table** **6 - The summary of sequence assembly of MSMs by SMARTdenovo.**

|  | Assembled contig of MSM 1 | | Assembled contig of MSM 2 | | Assembled contig of MSM 3 | |
| --- | --- | --- | --- | --- | --- | --- |
|  | Length (bp) | Number | Length (bp) | Number | Length (bp) | Number |
| N90 | 6,058 | 34 | 5,683 | 55 | 6,342 | 49 |
| N80 | 8,631 | 28 | 8,072 | 45 | 8,761 | 39 |
| N70 | 10,071 | 23 | 9,658 | 37 | 11,298 | 32 |
| N60 | 10,531 | 19 | 11,342 | 30 | 13,685 | 26 |
| N50 | 12,698 | 15 | 12,035 | 24 | 14,612 | 21 |
| N40 | 13,653 | 11 | 13,529 | 18 | 15,060 | 16 |
| N30 | 14,498 | 8 | 14,219 | 13 | 17,274 | 11 |
| N20 | 16,877 | 5 | 15,450 | 8 | 20,233 | 7 |
| N10 | 23,015 | 2 | 19,023 | 4 | 21,062 | 4 |
| Longest contigs | 23,054 |  | 33,154 |  | 28,057 |  |
| Total_size | 458,342 |  | 735,447 |  | 741,194 |  |
| Assembled contigs | >= 100 bp | 45 | >= 100 bp | 71 | >= 100 bp | 64 |
| Assembled contigs | >= 2 kb | 45 | >= 2 kb | 71 | >= 2 kb | 64 |
| GC rate (%) | 39.1 | | 39.3 | | 39.3 | |
