# Supplementary material for: Genomic anatomy of male-specific microchromosomes in a gynogenetic fish
Source: PLoS Genet. 2021 Sep 7;17(9):e1009760. doi: 10.1371/journal.pgen.1009760 (PMC8448357; doi:10.1371/journal.pgen.1009760)
Supplement: S7 Table — (DOCX) [file pgen.1009760.s016.docx]

**Supplementary Table** **7 - PacBio sequencing summary of female and male gonads.**

| **Sample** | **Technical platform** | **Total reads** | **Data size (Gb)** | **Mean length**  **(bp)** | **N50 length**  **(bp)** | **Annotation ratio (%)** | **Total isoforms** |
| --- | --- | --- | --- | --- | --- | --- | --- |
| Female gonads | PacBio | 12,292,888 | 24.92 | 2,027 | 2,934 |  |  |
|  |  |  |  |  |  | 99.58 | 169,010 |
| Male gonads | PacBio | 10,057,952 | 19.10 | 1,898 | 3,877 |  |  |
