# Supplementary material for: Genomic anatomy of male-specific microchromosomes in a gynogenetic fish
Source: PLoS Genet. 2021 Sep 7;17(9):e1009760. doi: 10.1371/journal.pgen.1009760 (PMC8448357; doi:10.1371/journal.pgen.1009760)
Supplement: S8 Table — (DOCX) [file pgen.1009760.s017.docx]

**Supplementary Table** **8 - Illumina sequencing summary of female and male gonads.**

| **Sample** | **Developmental stage** | **Total reads** | **Total map reads** | **Total map rate** | **Total transcript**  **number** |  |
| --- | --- | --- | --- | --- | --- | --- |
| Female | 18 dah | 48,999,724 | 41,135,268 | 83.95% | 116,456 |  |
|  | 22 dah | 48,544,582 | 41,117,261 | 84.70% | 116,391 |  |
|  | 26 dah | 48,908,688 | 40,745,828 | 83.31% | 116,900 |  |
|  | 30 dah | 49,255,266 | 41,320,243 | 83.89% | 116,436 |  |
|  | 35 dah | 49,585,020 | 41,686,126 | 84.07% | 116,398 |  |
|  | 40 dah | 49,004,446 | 40,845,206 | 83.35% | 117,020 |  |
|  | 47 dah | 49,740,050 | 41,602,578 | 83.64% | 117,269 |  |
|  | 58 dah | 49,479,190 | 41,384,395 | 83.64% | 120,529 |  |
|  | 70 dah | 49,337,390 | 43,821,470 | 88.82% | 107,795 |  |
| Male | 18 dah | 49,159,634 | 41,751,277 | 84.93% | 115,412 |  |
|  | 22 dah | 48,983,964 | 41,024,070 | 83.75% | 116,536 |  |
|  | 26 dah | 49,223,430 | 41,628,255 | 84.57% | 115,568 |  |
|  | 30 dah | 49,505,934 | 41,693,898 | 84.22% | 115,944 |  |
|  | 35 dah | 49,123,160 | 41,371,525 | 84.22% | 115,590 |  |
|  | 40 dah | 49,067,534 | 40,897,790 | 83.35% | 115,519 |  |
|  | 47 dah | 49,631,300 | 42,251,126 | 85.13% | 113,992 |  |
|  | 58 dah | 49,662,864 | 41,423,795 | 83.41% | 114,931 |  |
|  | 70 dah | 48,915,778 | 41,211,543 | 84.25% | 116,411 |  |
