# Supplementary material for: Genomic anatomy of male-specific microchromosomes in a gynogenetic fish
Source: PLoS Genet. 2021 Sep 7;17(9):e1009760. doi: 10.1371/journal.pgen.1009760 (PMC8448357; doi:10.1371/journal.pgen.1009760)
Supplement: S9 Table — # Red color indicates the gene fragments with a conserved coding sequence. (DOCX) [file pgen.1009760.s018.docx]

**Supplementary Table** **9 - Summary of 42 unique potential male-specific gene fragments.**

| **Fragment** | **Aligned reference transcript** | **Gene name** | **Length (bp)** | **Aligned length (bp)** | **Identity (%)** | **Gene description** | **Database** |
| --- | --- | --- | --- | --- | --- | --- | --- |
| 6384 | RXN38373.1 | tesmin | 929 | 687 | 45.83 | testis-expressed sequence 2 -like protein | non-redundant protein sequences^#^ |
| 4606 | ENSCCRG00015015163.1 | Uncharacterized | 660 | 603 | 83.36 | Uncharacterized | transcripts/splice variants |
| 297 | ENSCCRG00015006292.1 | *pex11b* | 609 | 609 | 87.26 | peroxisomal biogenesis factor 11 beta | transcripts/splice variants |
| 4613 | ENSCCRG00000051188.1 | *ftr99* | 584 | 171 | 88.89 | finTRIM family, member 99 | transcripts/splice variants |
| 9941 | BAE46430.1 | *rt* | 564 | 510 | 55% | reverse transcriptase | non-redundant protein sequences^#^ |
| 10004 | ENSCCRT00000003522.1 | *trpv4* | 479 | 68 | 85.92 | transient receptor potential cation channel, subfamily V, member 4 | transcripts/splice variants |
| 5773 | ENSCCRG00000010223.1 | *megf8* | 471 | 460 | 84.32 | ras-related protein Rab-8B-like | transcripts/splice variants |
| 5774 | ENSCCRG00015016107.1 | *cdh7b* | 463 | 449 | 83.76 | cadherin 7b | transcripts/splice variants |
| 911 | ENSCCRG00015003586.1 | *nfkbiaa* | 430 | 430 | 86.41 | nuclear factor of kappa light polypeptide gene enhancer in B-cells inhibitor | transcripts/splice variants |
| 1387 | ENSCCRG00015010969.1 | Uncharacterized | 418 | 85 | 85.88 | Uncharacterized | transcripts/splice variants |
| 5513 | ENSDARP00000065995-D2 | *tmem183a* | 417 | 295 | 86.44 | transmembrane protein 183A | coding sequences of *C. gibelio*^#^ |
| 4534 | CI01000028_02211495_02287691-D2 | *sptbn4l* | 411 | 50 | 90.00 | spectrin beta chain, non-erythrocytic 4-like | coding sequences of *C. gibelio*^#^ |
| 9732 | ENSXMAP00000020395-D33 | *tfr51* | 407 | 263 | 75.28 | finTRIM 51 protein | coding sequences of *C. gibelio*^#^ |
| 5412 | ENSCCRG00020052400.1 | *med1* | 396 | 268 | 82.09 | mediator complex subunit 1 | transcripts/splice variants |
| 4057 | CI01000029_05614085_05663509 | *dnmt3al* | 392 | 145 | 91.724 | DNA (cytosine-5)-methyltransferase 3A-like | coding sequences of *C. gibelio*^#^ |
| 6608 | ENSCARG00000028559.1 | *slc5a6a* | 390 | 390 | 88.31 | solute carrier family 5 member 6a | transcripts/splice variants |
| 9993 | ENSCARG00000008863.1 | *dcbld1l* | 390 | 390 | 88.78 | discoidin, CUB and LCCL domain-containing protein 1-like | transcripts/splice variants |
| 6394 | ENSCCRG00020050872.1 | *ccdc58* | 389 | 389 | 96.41 | coiled-coil domain containing 58 | transcripts/splice variants |
| 6398 | ENSCCRG00015011087 | Uncharacterized | 373 | 369 | 89.66 | Uncharacterized | transcripts/splice variants |
| 1995 | XP_016383117.1 | *llgl1l* | 365 | 114 | 84.00 | lethal(2) giant larvae protein homolog 1-like | non-redundant protein sequences^#^ |
| 2878 | ENSCCRG00015010969.1 | Uncharacterized | 354 | 52 | 86.54 | Uncharacterized | transcripts/splice variants |
| 3488 | ENSCCRG00015043938 | *gabrb3* | 339 | 339 | 85.43 | gamma-aminobutyric acid type A receptor beta3 | transcripts/splice variants |
| 4381 | ENSCCRG00015042210.1 | *lats1* | 337 | 337 | 80.45 | large tumor suppressor kinase 1 | transcripts/splice variants |
| 9769 | ENSCARG00000057373.1 | *rbm39b* | 336 | 334 | 87.68 | RNA-binding protein 39-like | transcripts/splice variants |
| 3056 | ENSCCRG00000039375.1 | *arih2* | 330 | 303 | 88.5 | ariadne RBR E3 ubiquitin protein ligase 2 | transcripts/splice variants |
| 1394 | ENSCCRG00015010969.1 | Uncharacterized | 326 | 90 | 92.22 | Uncharacterized | transcripts/splice variants |
| 1226 | ENSCARG00000068815.1 | *stab1* | 319 | 181 | 86.19 | stabilin-1-like | transcripts/splice variants |
| 806 | ENSCARG00000012596.1 | Uncharacterized | 311 | 311 | 90.94 | Uncharacterized | transcripts/splice variants |
| 9881 | ENSORLP00000013561-D7 | *nrxn1al* | 300 | 197 | 73.09 | neurexin-1a-like | coding sequences of *C. gibelio*^#^ |
| 9844 | ENSCARG00000010774.1 | *mpeg1* | 297 | 297 | 93.07 | macrophage-expressed gene 1 protein-like | transcripts/splice variants |
| 4388 | ENSCCRG00015015163 | Uncharacterized | 297 | 297 | 84.14 | Uncharacterized | transcripts/splice variants |
| 9934 | ENSCARG00000018439.1 | *bmp6* | 296 | 292 | 90.4 | probable enoyl-CoA hydratase | transcripts/splice variants |
| 3576 | ENSORLT00000012467.2 | *lepr* | 295 | 24 | 95.83 | leptin receptor | transcripts/splice variants |
| 5328 | ENSDARP00000108328-D2 | *setd1a* | 287 | 287 | 92.64 | SET domain containing 1A | coding sequences of *C. gibelio*^#^ |
| 8296 | ENSCART00000010084.1 | Uncharacterized | 283 | 36 | 91.67 | Uncharacterized | transcripts/splice variants |
| 261 | ENSCCRG00000035360.1 | *cabp5b* | 277 | 277 | 88.89 | calcium binding protein 5b | transcripts/splice variants |
| 4071 | ENSCARG00000025563.1 | *ly6m4* | 257 | 252 | 84.7 | lymphocyte antigen 6 family member M4 | transcripts/splice variants |
| 264 | ENSCARG00000029943.1 | *trim16l* | 241 | 214 | 96.26 | tripartite motif-containing protein 16-like | coding sequences of *C. gibelio*^#^ |
| 10067 | ENSCARG00000055972.1 | *pnn* | 237 | 120 | 92.5 | pinin, desmosome associated protein | transcripts/splice variants |
| 1828 | ENSCCRG00015047474 | *gars1* | 226 | 226 | 87.71 | glycyl-tRNA synthetase 1 | transcripts/splice variants |
| 9813 | ENSCCRG00015037571 | *layna* | 209 | 209 | 92.06 | layilin a | transcripts/splice variants |
| 10071 | ENSCCRG00000038021.1 | *eif4enif1* | 206 | 206 | 82.04 | eukaryotic translation initiation factor 4E nuclear import factor 1 | transcripts/splice variants |

**#** Red color indicates the gene fragments with a conserved coding sequence.
