# Supplementary material for: Genomic anatomy of male-specific microchromosomes in a gynogenetic fish
Source: PLoS Genet. 2021 Sep 7;17(9):e1009760. doi: 10.1371/journal.pgen.1009760 (PMC8448357; doi:10.1371/journal.pgen.1009760)
Supplement: S10 Table — (DOCX) [file pgen.1009760.s019.docx]

**Supplementary Table** **10 -** **PacBio sequencing summary of female and male gonads without MSMs.**

| **Sample** | **Technical platform** | **Total reads** | **Data size (Gb)** | **Mean length**  **(bp)** | **N50 length**  **(bp)** | **Annotation ratio (%)** | **Total isoforms** |
| --- | --- | --- | --- | --- | --- | --- | --- |
| Gonads_1(0-5K) | PacBio | 10,911,342 | 15.12 | 1,385 | 1,553 |  |  |
|  |  |  |  |  |  | 99.51 | 123,210 |
| Gonads_2(4.5-10K) | PacBio | 2,259,041 | 11.18 | 4,950 | 5,721 |  |  |
