# Supplementary material for: Genomic anatomy of male-specific microchromosomes in a gynogenetic fish
Source: PLoS Genet. 2021 Sep 7;17(9):e1009760. doi: 10.1371/journal.pgen.1009760 (PMC8448357; doi:10.1371/journal.pgen.1009760)
Supplement: S11 Table — (DOCX) [file pgen.1009760.s020.docx]

**Supplementary Table 11 -** **Primers that used in this study.**

| **Primers** | **Sequence (5’-3’)** |
| --- | --- |
| *Cg*-M-s-F | CAACAAAAGGAGGCCATTAAC |
| *Cg*-M-s-R | TGCATGAAGAGACCTACCCTG |
| 6384-F (*tesmin*) | CCGATCCCGCAGCTGAATCCTC |
| 6384-R (*tesmin*) | ACAGGCAAGGGATATTGTGGC |
| 4606-F (Uncharacterized) | ATAAGCAAGCAGTAGGCGGCAG |
| 4606-R (Uncharacterized) | AATCCTAGTCTAGCTCCATGC |
| 297-F (*pex11b*) | ATGCTTCCTTCTGCTGACCAG |
| 297-R (*pex11b*) | GGTCTGGTGAGTTTGCTGGCC |
| 4613-F (*ftr99*) | GCTCCATGCTCAATCTGACTCC |
| 4613-R (*ftr99*) | GAGACATTAGGGTCCCCAGTG |
| 9941-F (*rt*) | CACCTTCAACTCAACCTTGCC |
| 9941-R (*rt*) | TGTTGAATAAAGCAAATCTGC |
| 10004-F (*trpv4*) | GAGTATCTTGGAGAGTGAGG |
| 10004-R (*trpv4*) | GTTGAATAAAGCAAATCTGG |
| 5773-F (*megf8*) | AGTGTGTTCCCCGAAGTGTCC |
| 5773-R (*megf8*) | GTGAACATATTCCTGACCA |
| 5774-F (*cdh7b*) | TCATTTGCATAGAACGTGTTG |
| 5774-R (*cdh7b*) | AAGTGTGTTCCCCGAAGTGTC |
| 911-F (*nfkbiaa*) | CTTCACAAGGAGAGTAAGACAC |
| 911-R (*nfkbiaa*) | GGGGCTCTTGATGCCTTGA |
| 1387-F (Uncharacterized) | CTGAGCACCATCACCTTGTC |
| 1387-R (Uncharacterized) | GGGAGTTTACTTAGCCTAAT |
| 5513-F (*tmem183a*) | CCACAGAGTAATTTCACTTC |
| 5513-R (*tmem183a*) | GCACACAACCTCGATAAACGTCCG |
| 4534-F (*sptbn4l*) | CAGTTGTGCAGACGACAATCAC |
| 4534-R (*sptbn4l*) | CTGTAGCCAAATTCCTTGACAC |
| 9732-F (*tfr51*) | ACACTGGTGTTGGTCCATTGTG |
| 9732-R (*tfr51*) | CTTCAGGTCTGGTGAGTTTGC |
| 5412-F (*med1*) | AACTGCAAGTGACCATAATTGT |
| 5412-R (*med1*) | AAAGACTGTGGGGCTGGTCACC |
| 4057-F (*dnmt3al*) | TACTGCTTCAATTTGGCGC |
| 4057-R (*dnmt3al*) | AGTCACTGCACTCGTTTAC |
| 6608-F (*slc5a6a*) | GCCTCCACAGTCACCGGACC |
| 6608-R (*slc5a6a*) | GCTTCAAGAGGTAGTCACCTG |
| 9993-F (*dcbld1l*) | CATGACTATGGAAATTGTAGC |
| 9993-R (*dcbld1l*) | TCACCGGACCTGAACCCAA |
| 6394-F (*ccdc58*) | CCTTGAAGTTCTTGATGATCC |
| 6394-R (*ccdc58*) | CATTATGTCAGGCTGATTGGG |
| 6398-F (Uncharacterized) | CAAGCTCTGCACTGGTGGCAC |
| 6398-R (Uncharacterized) | TTCTTCCCATTGTTAACCATG |
| 1995-F (*llgl1l*) | TTACAATGGGAATGTTGCAC |
| 1995-R (*llgl1l*) | AGTCACCTCTCAAATGTACCAT |
| 2878-F (Uncharacterized) | GGGAAATGGAAGAGAGTGAG |
| 2878-R (Uncharacterized) | TCTGACGTAATTGTTCATGCC |
| 3488-F (*gabrb3*) | TGTATTCAAAGCATGTTAACAG |
| 3488-R (*gabrb3*) | CCATTCCTTGTGAAGTTCACT |
| 4381-F (*lats1*) | CCTTTAGGGGTCTTTGAGAATG |
| 4381-R (*lats1*) | TACAGAGTATGGAGCATATGA |
| 9769-F (*rbm39b*) | CTGAAGGCATCAAAACTATGA |
| 9769-R (*rbm39b*) | CGAGATGGTTTAGGGGTGAG |
| 3056-F (*arih2*) | AAGGTCCTAGAGTCTTGAGGAG |
| 3056-R (*arih2*) | TAAGTGCTCCAAAATTTCTTG |
| 1394-F (Uncharacterized) | CTGGAACAATACTATGTCTGTG |
| 1394-R (Uncharacterized) | CAAGTGTTATCCATCCTCATA |
| 1226-F (*stab1*) | ACAACCAAGCAGAGAACACG |
| 1226-R (*stab1*) | GCCTTCAAAATGGTCTCTCA |
| 806-F (Uncharacterized) | TCATCTCTAATAGCTGACGAC |
| 806-R (Uncharacterized) | GATAGGATCAAGCGGGCAAGT |
| 9881-F (*nrxn1al*) | TTCAGGTCTGGTGAGTTTGCTG |
| 9881-R (*nrxn1al*) | AGTTTCCACAGTCTGTGATG |
| 9844-F (*mpeg1*) | GAAGAAGGAGGCGGGAACCG |
| 9844-R (*mpeg1*) | AGAGGCGTGGGAACGAGGAG |
| 4388-F (Uncharacterized) | TACAGAGTATGGAGCATATGA |
| 4388-R (Uncharacterized) | CCTTTAGGGGTCTTTGAGAAT |
| 9934-F (*bmp6*) | CGAGAGGCGTGGGAACGAGGA |
| 9934-R (*bmp6*) | GGCCTGGTCCTCTCTCGTCCTTC |
| 3576-F (*lepr*) | GGGACTCAATTCAGGTAGA |
| 3576-R (*lepr*) | AGACTGAACTGAACTCATACC |
| 5328-F (*setd1a*) | GTGAGGATGAGCACGGAAGT |
| 5328-R (*setd1a*) | ATGGGAGCCGAGAAAGAGAG |
| 8296-F (Uncharacterized) | TCTATAGCTGACGACTTTGCA |
| 8296-R (Uncharacterized) | TTGAAGGAGATGAGATGGAAT |
| 261-F (*capb5b*) | CAGGTCTGGTGAGTTTGCTGGC |
| 261-R (*capb5b*) | TGGTGTTGGTCCATTGTG |
| 4071-F (*ly6m4*) | TTGGCATTCTCTTGATGAGC |
| 4071-R (*ly6m4*) | ACAGTCACCGGACCTGAACCC |
| 264-F (*trim16l*) | TGCTTTTGGCAGTGTACCAT |
| 264-R (*trim16l*) | CATTTAACCAACGTTTGGTG |
| 10067-F (*pnn*) | CAAGGTTTTATTGAATGCTAT |
| 10067-R (*pnn*) | TCGATGCATCGATTAATCGTA |
| 1828-F (*gars1*) | TCTTGGTAAACGAGCTGCAGTG |
| 1828-R (*gars1*) | GTTTTGTATTTTCATTTGGAA |
| 9813-F (*layna*) | ATCAAGCACACCAACACCAT |
| 9813-R (*layna*) | AAAACCAAAGCACTGCACTC |
| 10071-F (*eif4enif1*) | AATACTTTCTGTTTGCCGT |
| 10071-R (*eif4enif1*) | ATTCACACAGACCGACCTGT |
| *β-actin*-F | AGCACGGTATTGTGACTAACTG |
| *β-actin*-R | TCGAACATGATCTGTGTCATC |
