# Supplementary material for: Genomic anatomy of male-specific microchromosomes in a gynogenetic fish
Source: PLoS Genet. 2021 Sep 7;17(9):e1009760. doi: 10.1371/journal.pgen.1009760 (PMC8448357; doi:10.1371/journal.pgen.1009760)
Supplement: Attachment — Submitted filename: Review on manuscript Plosgenetics 10032021.docx [file pgen.1009760.s021.docx]

Review on manuscript entitled « genomic anatomy of male-specific microchromosomes reveals an evolutionary mechanism of sex chromosome in a gynogenetic fish » by Ding and collaborators.

This manuscript reports on the characterization of the genomic content of some male specific microchromosomes (MSMs) in the gibel carp (Carassius gibelio) after microdissection and sequencing. The question on the potential implication of these MSMs in gibel carp sex determination is indeed a very interesting and important question. To answer this question Ding and collaborators first characterized a male-specific repetitive element highly enriched in some MSMs. MSMs were then labelled using this male-specific repetitive element, micro-dissected, amplified and sequenced with a long-reads sequencing approach. Based on these sequences Ding and collaborators explored the structure and gene content of MSMs and found that they are slightly enriched for some repetitive elements and also contains sequences derived from all chromosomes A. They also characterized MSM genes (or gene fragments?) with a male-restricted or male-biased differential expression.

The overall strategy is interesting with some really challenging approaches (specific labelling of MSMs, microdissection and sequencing after multiple displacement amplification) but unfortunately this manuscript suffers from major problems including for instance the lack of important information and/or results, complicated and sometime uncomplete analyses, and no real discussion with too many conclusions not supported by the results (see major points below).

**MAJOR POINTS**

Lack of important information and/or results:

It is not mentioned in the manuscript why only 3 MSMs were retained among the 4 MSMs. It is indeed important as this remaining unexplored MSM may contains key information for sex determination. In the text lines 189-191, it is mentioned “Three male-specific samples were screened, and the presence of the marker indicated that they were MSMs. I do not understand how these samples were characterized as male-specific samples before being screened with the male-specific marker. In addition, the overall strategy here is not well explained as it is unclear from the text whether the Cg-Ca-CL1 repetitive element is present in all microchromosomes or just the MSMs. The fact that Cg-Ca-CL1 shares the same consensus sequence as the previously identified Cg-M-s male-specific sequence would suggest that Cg-Ca-CL1 is also MSM-specific but later in the text we can read that Cg-Ca-CL1 can be used to identify microchromosomes suggesting that Cg-Ca-CL1 is not restricted to MSMs. The use of a male-specific marker derived from the sequence of Cg-M-s is also confusing as from the text we understand that both Cg-Ca-CL1 and Cg-M-s share the same core sequence. So, what is the male-specificity of Cg-M-s compared to Cg-Ca-CL1?

PacBio is not a sequencing technology (just the name of a biotechnology company) and it is not mentioned in the text (results and M&M) what kind of PacBio technology was used to sequence amplified products of MSMs, i.e., CLR or HiFi technology. CLR produces uncorrected long-reads with an average error rate of ~ 15%, and Hifi produced corrected long reads (99.9% accuracy). This read accuracy could be important here as microchromosome sequences being derived from A chromosome segments, successful remapping depends whether they share high homologies with their A counterparts. With 15 % error rates CLR reads would be less informative than HiFi reads even if long fragment matches may counterbalance these local lacks of identity. Surprisingly also, no MSM sequence assembly is reported in the result section. This would seem to be the first thing to do to enable a real genomic investigation of the structure (anatomy?) of these MSMs. With CLR reads such MSM assemblies would have to be corrected by a layer of accurate short-read sequencing. Important information could be deduced from these MSM assemblies including for instance sequence divergence of the MSM segments compared to their A chromosomes sequences, their precise gene annotation, comparison of the sequence, genes of these different MSMs (are they very different in their gene content? Structure? Do their share some redundancy in their gene content? …), and much more …

MSM gene names are not provided in the manuscript (only mentioned as anonymous gene ID like Isoform_38102). This is really not a good communication strategy as I honestly doubt that the authors of this manuscript did not look at that important information. As a reviewer (and probably as a simple reader also) being left with no-name genes with a sex-biased expression being on an MSM is very frustrating as we all know that it is really not complicated to provide homology annotation that would bring a lot of important information to this manuscript (are some of these gene potentially implicated in the differentiation of the gonad, being potential good candidate as master gene for sex determination?).

Complicated and/or sometime uncomplete analyses:

The identification of the gene content of MSMs is very complicated to follow as we jump from different transcript to gene numbers depending on what transcriptomic or in silico dataset were used to annotate these transcripts, genes. Later in the following paragraph on gene expression another complexity popup with the undefined terms of “transcript fragments” and “gene fragments”. Apart from being complicated to follow this unexplained terminology also suggest that not all these transcripts and genes are complete (with regards to their A chromosome counterparts). This is probably not completely unexpected as some genes and their CDS may have been duplicated as partial truncated genes on the MSMs (see for instance the recent paper of Ahmad et al., 2020). This paragraph needs to be revised and simplified (may be just reports gene numbers for simplicity in the main text) and the “gene fragments” term needs to be explained. The gene contents of the different MSMs need to be also compared as it is not complicated to do and would bring interesting information.

After a rather complicated subtraction strategy (this paragraph would also need some simplification) to characterize MSM genes with a male-specific or male-predominant expression we are left with a single sentence stating that “Eventually, five and three gene fragments (among 50 unique gene fragments with a potentially sex-biased expression) are confirmed to have male-specific and male-biased expression in gonads, respectively, during the developmental period of sex determination (Fig 6F and 6G), which may play important roles in male determination or differentiation.”. No indication is given on the rationale behind this stringent choice (7 among 50).

No real discussion with too many conclusions not supported by the results:

The final discussion is very short which could be a good point. But this manuscript raised many interesting questions and many results would need a real detailed discussion Instead this short discussion contains many conclusions that are not well supported by the results.

One example is for instance: “Thus, the MSMs responsible for male determination have been selected and preserved, facilitating male occurrence and the evolutionary transition of the reproduction mode from unisexual gynogenesis to sexual reproduction in the unisexual gynogenetic C. gibelio”. No other evidence apart from the correlation between these MSMs and the normal (WT) male phenotype (already published information) is provided in the manuscript. So, the role of these MSMs in the sex determination process of gibel carp remains unclear. The identification of potential candidate gene known to be involved in sex differentiation or sex determination would have been another interesting evidence that these MSMs could have a sex determining role (along with the precise characterization of its gonadal gene expression pattern during early developmental stage). Ultimately functional inactivation of such a MSM sex determining candidate gene would provide another important evidence of such a role of MSMs. In absence of these new evidence we cannot say that the MSMs are responsible for male determination. The end of the sentence is also not supported by any results.

The final conclusion sentence “Based on previous findings and this study, we present a noncanonical mechanism for sex chromosome evolution, in which supernumerary chromosomes participate in the process of sex chromosome evolution via evolving into or providing materials for sex chromosomes” is also not supported by the results as no (additional) evidence are provided to support a role of MSMs in the process of sex chromosome evolution.

**SOME MINORS POINTS**

Results lines 148: It could be interesting to provide again the information why this repetitive sequence characterization is important in the context of this study. Starting directly with “To determine the repetitive sequences ….” may be difficult for readers that would have read too quickly the introduction or would not have read previous papers on this MSMs in gibel carp. The comparison with common carp is also not explained and we only understand later (if I understood well) in the text that it was used to find MSM repetitive elements as these MSMs are not found in common carp. Starting with an explanation of what is done and why would help the reader to better understand this first paragraph of the results.

Results lines 223, 226 and 231: conclusion of this paragraph that that some repetitive elements have been extensively accumulated on the MSMs is exaggerated as there is only a 1,2- to 1.5-fold change between the whole genome assembly (including microchromosomes?) and the sequences from MSMs. An extensive accumulation would probably be something much higher.

Quantitative RT-QPCR should be used instead of an end-point classical RT-PCR that is not an accurate way of measuring gene expression.

**REFERENCE**

Ahmad, S.F., Jehangir, M., Cardoso, A.L. *et al.* B chromosomes of multiple species have intense evolutionary dynamics and accumulated genes related to important biological processes. *BMC Genomics* **21,** 656 (2020). https://doi.org/10.1186/s12864-020-07072-1
