# Supplementary material for: Genomic anatomy of male-specific microchromosomes in a gynogenetic fish
Source: PLoS Genet. 2021 Sep 7;17(9):e1009760. doi: 10.1371/journal.pgen.1009760 (PMC8448357; doi:10.1371/journal.pgen.1009760)
Supplement: Attachment — Submitted filename: Response to Reviews.docx [file pgen.1009760.s022.docx]

Dear Editor Manfred Schartl, Editor-in-Chief Gregory Copenhaver, and reviewers,

Thanks for your efforts in reviewing and processing the manuscript entitled 'Genomic anatomy of male-specific microchromosomes reveals an evolutionary mechanism of sex chromosome in a gynogenetic fish' (PGENETICS-D-21-00204). According to the valuable comments and suggestions of reviewers, we have done numerous revisions through the whole manuscript such as modifying the title, revising several paragraphs, adding new data, deleting uncertain results, correcting some mistakes, and rewriting the discussion section. And all the revisions are marked with red color for easy checking. Responses to each comment are listed as following.

**Reviewer #1:**

I was turned off by the very first sentence of this paper, and suspected the entire premise was wrong. I was forced to read, with considerable skepticism, the many previous papers by these authors. I was eventually convinced that they have identified some microchromosomes that contribute to male sex determination in this gynogenetic species. I have yet to see evidence that any sexual reproduction is occurring in this system. I believe these microchromosomes are simply a genomic parasite on the gynogenetic system. This is of course interesting, but represents a very different spin on the interpretation of the results.

**Response:** We are very sorry about your tough review experience on the original manuscript, and thank you very much for your effort to read our previous papers. We have changed the arbitrary premise that unisexual taxa evolve into sexual organisms eventually, and revised the title, abstract, introduction, results, and discussion accordingly. Besides, we also lay out the hypothesis in the discussion section that these microchromosomes might be a genomic parasite in gynogenetic gibel carp.

Abstract

There is so much wrong with this first sentence. What evidence is there that ‘unisexual’ taxa evolve (not develop!) into sexual organisms? I was under the impression that gynogenetic taxa evolve from sexual (or dioecious) taxa, and eventually go extinct. It is entirely inappropriate to describe any of this as a “long-term evolutionary strategy”.

**Response:** Thanks for pointing out these mistakes, and we have deleted these sentences and rewritten the whole premise in the abstract (line 23-31 in the revised manuscript).

Introduction

Avise (ref 3) DOES NOT suggest that gynogenetic forms evolve into sexual species: “In summation, molecular, as well as ecological and distributional, data suggest that unisexual clonality in vertebrate animals can best be viewed as a genetic operation that sometimes offers a highly successful tactic in the ecological short term but that almost invariably fails as a long-term evolutionary strategy.”

**Response:** Thank you for pointing out our misinterpretation about the paper of Avise. We have corrected the wrong description and rewritten the whole paragraph in the introduction section (line 94-111 in the revised manuscript).

Results

Figure 1A – Why are the two clouds of dots different colors (black and blue)?

**Response:** Thanks for your comments. In Figure 1A of original manuscript, the blue cloud indicates the centromere repeats and the black cloud indicates the rest repetitive sequences. To avoid ambiguity, we have changed all the dots into black and highlighted the repetitive sequences expanded in *C. gibelio* using the black ellipse (Figure 1 A and line 152-154 in the revised manuscript).

Figure 2E-G – What exactly am I supposed to conclude from these figures? I do not see evidence of colocalization of green and red signals.

**Response:** Thanks for your comments. We are very sorry that we did not present the overlapped image clearly, as the red fluorescence generated by microchromosome-specific PNA probes is too strong, which has covered up the green signals generated by amplified DNA probes. We have added arrows to indicate the microchromosomes with intensive signals generated by amplified DNA probes and revised the corresponding description (Figure 2E-G and line 203-206 in the revised manuscript).

Discussion

Line 326 should also cite for example of B chr evolving into sex chromosome. Clark FE, Kocher TD. Changing sex for selfish gain: B chromosomes of Lake Malawi cichlid fish. Sci Rep. 2019 Dec 27;9(1):20213

**Response:** Thanks for your suggestion. We have cited this article in the discussion section (line 356 and 392 in the revised manuscript).

I think you should be more explicit about a model for this process. Gynogenetic carp seem to occasionally pick up DNA/chromosomes from the sperm donor. It appears that you may have identified a selfish supernumerary chromosome(s) that has exploited this process for its own reproduction. It may further carry a male sex determiner to further promote its transmission. It is still surprising to me that this seems to require the transmission of 3 microchromosomes. I would have expected the required elements to be closely linked on a single chromosome. But perhaps there is a mechanism that maintains linkage among the three microchromosomes. I do not see any evidence to support your idea (Figure 7) that these microchromosomes arose from with the hexaploidy gynogen. Instead, I suspect they arose from sperm DNA. Perhaps you can lay out several explicit hypotheses for the origins of these microchromosomes and evaluate their likelihood using all the sources of evidence available.

**Response:** Thank you for your comments and suggestions. We have proposed several hypotheses for the origin and evolution of these microchromosomes and revised the Figure 7. Besides, based on the available data in *C. gibelio* and related studies in other taxa, we also evaluated the likelihood of these hypotheses (line 363-400 in the revised manuscript).

The analysis of repeat/gene content on the microchromosomes was a necessary exercise, but does not seem to have resulted in the identification of any particularly interesting candidates.

**Response:** Thanks for your comments. Our present results strongly indicate that MSMs are responsible for male determination in *C. gibelio*, and we have found a few MSM-linked genes with male-specific or male-biased transcriptional activity during developmental stage of sex determination. One gene with male-specific expression was annotated to be a ion channel gene *trpv4* (transient receptor potential cation channel, subfamily V, member 4), which was suggested to be a possible regulator of sox9 and play a male differentiation role in the American alligator (line 320-323 in the revised manuscript). These results provide a clue to further identify the sex-determining gene/genes on MSMs and continued investigations are being performed in our lab.

Materials and methods

How many fish were sampled in this study?

For the analysis in Figure 2C-D it appears to be 1 male (13 mChr) and 1 female (9 mChr)?

It is not clear there was any replication for Figure 2E-G.

The actual number of fish examined for each portion of the study needs to be made explicit.

**Response:** Thanks for your questions and suggestions, and we have added the actual number of sampled fish for each experiment (line 420, 438, 454-455, 466, 509, 511, 513, and 543 in the revised manuscript).

For the analysis in Figure 2C-D, one male metaphase cell and one female metaphase cell were used (line 184-188 and 190-194 in the revised manuscript).

For the analysis in Figure 2E-G, a total of three males were used as replication for co-localization of amplified DNAs and microchromosome-specific PNA probes (line 466-467 in the revised manuscript).

**Reviewer #2:**

This manuscript reports on the characterization of the genomic content of some male specific microchromosomes (MSMs) in the gibel carp (*Carassius gibelio*) after microdissection and sequencing. The question on the potential implication of these MSMs in gibel carp sex determination is indeed a very interesting and important question. To answer this question Ding and collaborators first characterized a male-specific repetitive element highly enriched in some MSMs. MSMs were then labelled using this male-specific repetitive element, micro-dissected, amplified and sequenced with a long-reads sequencing approach. Based on these sequences Ding and collaborators explored the structure and gene content of MSMs and found that they are slightly enriched for some repetitive elements and also contains sequences derived from all chromosomes A. They also characterized MSM genes (or gene fragments?) with a male-restricted or male-biased differential expression.

The overall strategy is interesting with some really challenging approaches (specific labelling of MSMs, microdissection and sequencing after multiple displacement amplification) but unfortunately this manuscript suffers from major problems including for instance the lack of important information and/or results, complicated and sometime uncomplete analyses, and no real discussion with too many conclusions not supported by the results (see major points below).

**Response:** Thanks for your valuable comments and suggenstions. We have done numerous revisions throughout the manuscript such as adding the lacking information, performing sequence assembly, providing new data, revising several paragraphs, correcting some mistakes, and rewriting the whole discussion section (see the responses to all the major and minor points).

**MAJOR POINTS**

Lack of important information and/or results:

It is not mentioned in the manuscript why only 3 MSMs were retained among the 4 MSMs. It is indeed important as this remaining unexplored MSM may contains key information for sex determination. In the text lines 189-191, it is mentioned “Three male-specific samples were screened, and the presence of the marker indicated that they were MSMs.” I do not understand how these samples were characterized as male-specific samples before being screened with the male-specific marker. In addition, the overall strategy here is not well explained as it is unclear from the text whether the Cg-Ca-CL1 repetitive element is present in all microchromosomes or just the MSMs. The fact that Cg-Ca-CL1 shares the same consensus sequence as the previously identified Cg-M-s male-specific sequence would suggest that Cg-Ca-CL1 is also MSM-specific but later in the text we can read that Cg-Ca-CL1 can be used to identify microchromosomes suggesting that Cg-Ca-CL1 is not restricted to MSMs. The use of a male-specific marker derived from the sequence of Cg-M-s is also confusing as from the text we understand that both Cg-Ca-CL1 and Cg-M-s share the same core sequence. So, what is the male-specificity of Cg-M-s compared to Cg-Ca-CL1?

**Response:** According to your comments, we have added the information why only 3 MSMs were retained among the 4 extra microchromosomes (line 195-200 in the revised manuscript). One male metaphase cell containing 13 microchromosomes and one female metaphase cell containing 9 microchromosomes were used for fluorescence microdissection, multiple displacement amplification (MDA), and PCR detection with male-specific marker. The male metaphase cell had 4 extra microchromosomes than the female metaphase cell, but only 3 extra microchromosomes were identified as MSMs (Figure 2A-D), which might be caused by two main reasons. The first reason was that the male metaphase truly had 1 extra microchromosomes without male specificity, and the other explanation is that the MDA-based DNA amplification of a single microchromosome cannot amplify the entire DNA.

We have rewritten this paragraph and changed this sentence “Three male-specific samples were screened, and the presence of the marker indicated that they were MSMs.” (line 188-195 in the revised manuscript).

To make the relationship between *Cg*-M-s and *Cg*-*Ca*-CL1 be more explicit, we have added a supplementary figure (Supplementary Figure S1) and corresponding description (line 159-160 and line 164-166) in the revised manuscript.

PacBio is not a sequencing technology (just the name of a biotechnology company) and it is not mentioned in the text (results and M&M) what kind of PacBio technology was used to sequence amplified products of MSMs, i.e., CLR or HiFi technology. CLR produces uncorrected long-reads with an average error rate of ~ 15%, and Hifi produced corrected long reads (99.9% accuracy). This read accuracy could be important here as microchromosome sequences being derived from A chromosome segments, successful remapping depends whether they share high homologies with their A counterparts. With 15 % error rates CLR reads would be less informative than HiFi reads even if long fragment matches may counterbalance these local lacks of identity. Surprisingly also, no MSM sequence assembly is reported in the result section. This would seem to be the first thing to do to enable a real genomic investigation of the structure (anatomy?) of these MSMs. With CLR reads such MSM assemblies would have to be corrected by a layer of accurate short-read sequencing. Important information could be deduced from these MSM assemblies including for instance sequence divergence of the MSM segments compared to their A chromosomes sequences, their precise gene annotation, comparison of the sequence, genes of these different MSMs (are they very different in their gene content? Structure? Do their share some redundancy in their gene content? …), and much more …

**Response:** Thanks for your comments and suggestions. The amplified products of three microdissected MSMs were sequenced via the continuous long-read (CLR) of Sequel II (PacBio platform), which has been added in the revised manuscript (line 212 and 472-473 in the revised manuscript).

We have provided the data of sequence assembly and corresponding analyses in the revised manuscript. The sequencing reads of each MSM were self-corrected by CANU (Supplementary Table S3) and then assembled via CANU (Supplementary Table S4), SPAdes (Supplementary Table S5), and SMARTdenovo (Supplementary Table S6), respectively. Subsequently, the genome assembly of a female *C. gibelio* and the full-length gonadal transcriptomes were used as references respectively to assess the assemblies of MSMs. All the contigs and corrected reads displayed much lower alignment level to the references in sharp contrast with the clean reads (Supplementary Figure S6), which might be caused by removal of repetitive sequences during the process of correcting and assembling. Thus, to better reflect the reality of sequence composition, we only used the clean reads for subsequent analyses (line 215-224 and 475-491).

According to your advices, we have added the sequence divergence of MSM segments compared to their corresponding A chromosome sequences (Figure 3D and line 231-236 in the revised manuscript). Pariwise comparative analysis also performed among these three MSMs (Figure 3E and line 236-240 in the revised manuscript). Besides, gene content of these MSMs have been analyzed in the revised manuscript (Supplementary Figure S7 and line 268-271). However, it is hard to obtain gene structure information of MSMs, as most genes on MSMs are fragmental.

MSM gene names are not provided in the manuscript (only mentioned as anonymous gene ID like Isoform_38102). This is really not a good communication strategy as I honestly doubt that the authors of this manuscript did not look at that important information. As a reviewer (and probably as a simple reader also) being left with no-name genes with a sex-biased expression being on an MSM is very frustrating as we all know that it is really not complicated to provide homology annotation that would bring a lot of important information to this manuscript (are some of these gene potentially implicated in the differentiation of the gonad, being potential good candidate as master gene for sex determination?).

**Response**: We are sorry about the rough results in the original manuscript. We have added the annotation information of gene fragments in the Supplementary Table S8 in the revised manuscript. One gene with male-specific expression was annotated to be a ion channel gene *trpv4* (transient receptor potential cation channel, subfamily V, member 4), which was suggested to be a possible regulator of sox9 and play a male differentiation role in the American alligator (line 320-323 in the revised manuscript). These results provide a clue to further identify the sex-determining gene/genes on MSMs and continued investigations are being performed in our lab.

Complicated and/or sometime uncomplete analyses:

The identification of the gene content of MSMs is very complicated to follow as we jump from different transcript to gene numbers depending on what transcriptomic or in silico dataset were used to annotate these transcripts, genes. Later in the following paragraph on gene expression another complexity popup with the undefined terms of “transcript fragments” and “gene fragments”. Apart from being complicated to follow this unexplained terminology also suggest that not all these transcripts and genes are complete (with regards to their A chromosome counterparts). This is probably not completely unexpected as some genes and their CDS may have been duplicated as partial truncated genes on the MSMs (see for instance the recent paper of Ahmad et al., 2020). This paragraph needs to be revised and simplified (may be just reports gene numbers for simplicity in the main text) and the “gene fragments” term needs to be explained. The gene contents of the different MSMs need to be also compared as it is not complicated to do and would bring interesting information.

**Response**: Thank you for your comments and suggestions. We have rewritten this paragraph to make the annotation part more explicit (line 261-268 in the revised manuscript) and added the comparison of gene contents among the different MSMs (Figure 5C, Supplementary Figure S8, and line 268-271 in the revised manuscript). Besides, we have deleted the undefined term of “transcript fragments” and defined the term of “gene fragments” (line 298-301 in the revised manuscript).

After a rather complicated subtraction strategy (this paragraph would also need some simplification) to characterize MSM genes with a male-specific or male-predominant expression we are left with a single sentence stating that “Eventually, five and three gene fragments (among 50 unique gene fragments with a potentially sex-biased expression) are confirmed to have male-specific and male-biased expression in gonads, respectively, during the developmental period of sex determination (Fig 6F and 6G), which may play important roles in male determination or differentiation.”. No indication is given on the rationale behind this stringent choice (7 among 50).

**Response**: Thanks for your comments. We have simplified this paragraph (line 287-304 in the revised manuscript) and provided the results of qPCR analyses (Figure 6F and Supplementary Figure S9), which excluded most of these genes with similar expression pattern between females and males.

No real discussion with too many conclusions not supported by the results:

The final discussion is very short which could be a good point. But this manuscript raised many interesting questions and many results would need a real detailed discussion Instead this short discussion contains many conclusions that are not well supported by the results.

One example is for instance: “Thus, the MSMs responsible for male determination have been selected and preserved, facilitating male occurrence and the evolutionary transition of the reproduction mode from unisexual gynogenesis to sexual reproduction in the unisexual gynogenetic C. gibelio”. No other evidence apart from the correlation between these MSMs and the normal (WT) male phenotype (already published information) is provided in the manuscript. So, the role of these MSMs in the sex determination process of gibel carp remains unclear. The identification of potential candidate gene known to be involved in sex differentiation or sex determination would have been another interesting evidence that these MSMs could have a sex determining role (along with the precise characterization of its gonadal gene expression pattern during early developmental stage). Ultimately functional inactivation of such a MSM sex determining candidate gene would provide another important evidence of such a role of MSMs. In absence of these new evidence we cannot say that the MSMs are responsible for male determination. The end of the sentence is also not supported by any results.

The final conclusion sentence “Based on previous findings and this study, we present a noncanonical mechanism for sex chromosome evolution, in which supernumerary chromosomes participate in the process of sex chromosome evolution via evolving into or providing materials for sex chromosomes” is also not supported by the results as no (additional) evidence are provided to support a role of MSMs in the process of sex chromosome evolution.

**Response**: Thanks for pointing out the deficiency of discussion in the original manuscript. We have rewritten the whole discussion section (line 334-350 and 363-407 in the revised manuscript) and proposed several hypotheses for the origin and evolution of these MSMs, whose likelihood were evaluated based on the available data in this system.

**SOME MINORS POINTS**

Results lines 148: It could be interesting to provide again the information why this repetitive sequence characterization is important in the context of this study. Starting directly with “To determine the repetitive sequences ….” may be difficult for readers that would have read too quickly the introduction or would not have read previous papers on this MSMs in gibel carp. The comparison with common carp is also not explained and we only understand later (if I understood well) in the text that it was used to find MSM repetitive elements as these MSMs are not found in common carp. Starting with an explanation of what is done and why would help the reader to better understand this first paragraph of the results.

**Response:** Thank you for your valuable suggestion. We have provided some information of why and how we find the repetitive element of microchromosomes in gibel carp (line 144-150 in the revised manuscript), in order to make the first paragraph to be better understood.

Results lines 223, 226 and 231: conclusion of this paragraph that that some repetitive elements have been extensively accumulated on the MSMs is exaggerated as there is only a 1,2- to 1.5-fold change between the whole genome assembly (including microchromosomes?) and the sequences from MSMs. An extensive accumulation would probably be something much higher.

**Response:** Thank you for your comments. We have changed these exaggerated statements (line 251 and 257 in the revised manuscript) and added the missing information that the whole genome assembly is derived from a female individual containing microchromosomes (line 252 in the revised manuscript).

Quantitative RT-QPCR should be used instead of an end-point classical RT-PCR that is not an accurate way of measuring gene expression.

**Response:** Thank you for your suggestion and we have added the results of relative quantitative PCR (Figure 6F, Supplementary Figure S9, and line 314-325 in the revised manuscript).

**REFERENCE**

Ahmad, S.F., Jehangir, M., Cardoso, A.L. *et al.* B chromosomes of multiple species have intense evolutionary dynamics and accumulated genes related to important biological processes. *BMC Genomics* **21,** 656 (2020). https://doi.org/10.1186/s12864-020-07072-1

**Reviewer #3:**

Ding and coauthors continued with the analysis of gibel carps’s male extra microchromosomes. Microchromosomes were also reported in other fish species and most of these microchromosomes were considered to be supernumerary chromosomes, which were suggested to have an effect on the phenotype of host species, especially the sex. In order to check how these extra microchromosomes are related to male sex determination in gibel carp, the authors applied chromosome microdissection, whole genome amplification, next-generation sequencing and bioinformatic analysis to unravel their DNA composition.

Considering that the copy number of supernumerary chromosomes often exceeds two, I’m wondering whether the three analysed MSMs represent different types of MSMs or only one type. Taken the highly similar sequence composition (shown in figures 3 and 4) it seems to me that only one type of MSM exists in gibel carp. Small sequence differences between the three next generation sequence sets are likely caused by the fact that MDA-based DNA amplification of a single chromosome never amplifies 100% of their DNA. The technical limitations of single-copy chromosome microdissection/sequencing is likely also the reason why only three and not four MSMs showed a male-specific PCR product (in figure 2G). I suggest providing experimental evidence that three types of MSMs exist or rewriting the manuscript accordingly.

**Response:** Thank you for your comments. We have added the data of comparative analysis among these three MSMs, such as sequence divergence of MSM segments compared to their corresponding A chromosome sequences (Figure 3D and line 231-236 in the revised manuscript), sequence similarity (Figure 3E and line 236-240 in the revised manuscript), and gene contents (Supplementary Figure S7 and line 268-271).

The conclusion that MSMs accumulated mitochondria-derived DNA needs additional proof. Could it be a byproduct of the microisolation process? To provide proof, I would suggest a PCR experiment with primers specific for MSMs located mitochondrial DNA using genomic DNA of a female (negative control) and a male (positive control). Alternatively, FISH could be performed using labelled mitochondrial DNA. MSMs and mitochondria located FISH signals would proof that MSMs accumulated organellar DNA.

**Response:** Thank you for your suggestions. We have performed qPCR detection of mitochondria-derived DNAs between females and males, however there is no significant differences between sexes. These results indicate that the mitochondria-derived DNAs of MSM 2 may be byproducts of the microisolation process. Thus, we have deleted these results that MSMs accumulated mitochondira-derived DNA in the revised manuscript.

Page 5, lines 129-131

“In this study, we developed….”. This sentence is difficult to follow. I would suggest the following: “In this study, we analysed the sequence composition of microdissected FISH-labelled microchromosomes and identified three male-specific microchromosomes (MSMs) in hexaploidy C. gibelio.

**Response:** According to your suggestion, we have rewritten this sentence (line 129-130 in the revised manuscript).

Page 7, 184 – 186u

It is unclear to me whether the 13 microchromosomes in a male and 9 microchromsomes in a female were isolated each from one metaphase cell each. Or did you isolate the mircochromosomes from several male and female cells.

**Response:** We are sorry for the unclear description. The 13 microchromosomes were isolated from one male metaphase cell and the 9 microchromosomes were isolated from one female metaphase cell (line 184-188 in the revised manuscript).

With thanks for your consideration.

Kind regards,

Sincerely yours,

Xi-Yin Li, Ph.D

Institute of Hydrobiology

Chinese Academy of Sciences

Wuhan 430072, China

Tel: +86-27-68780707

Fax: +86-27-68780123

E-mail: lixiyin@ihb.ac.cn
