# Supplementary material for: Genomic anatomy of male-specific microchromosomes in a gynogenetic fish
Source: PLoS Genet. 2021 Sep 7;17(9):e1009760. doi: 10.1371/journal.pgen.1009760 (PMC8448357; doi:10.1371/journal.pgen.1009760)
Supplement: Attachment — Submitted filename: Response to Reviews.docx [file pgen.1009760.s023.docx]

Dear Editor Manfred Schartl, Editor-in-Chief Gregory Copenhaver, and reviewers,

Thanks for your efforts in reviewing and processing the manuscript entitled 'Genomic anatomy of male-specific microchromosomes in a gynogenetic fish' (PGENETICS-D-21-00204R1). According to the valuable comments and suggestions of reviewers, we have revised the whole manuscript carefully. And all the revisions are marked with red color for easy checking. Responses to each comment are listed as following.

**Reviewer #2:**

First, I would like to thanks the authors for the important modifications that have been made on their manuscript. Many of these modifications provide good and interesting answers to most of my comments.

However, I still have some concerns on the MSM gene annotation as it is unclear in this new revised manuscript what, and how many, genes are present on the MSMs with a potential open reading frame (intact genes). You are mentioning “gene fragments” but it would be important to know how many of them have a conserved CDS compared to their paralogous copy (or copies) in the A chromosome part of the genome. A more detailed annotation should be provided (not only a blast analysis).

**Response:** Thanks for your comments and suggestions. We performed analysis of gene integrity based on the integrity of potential coding sequences (line 626-629 in the revised manuscript). We found out that the majority annotated genes had the integrity scores less than 10%, which accounted for 78.95% (180 genes), 81.82% (72 genes), and 76.85% (156 genes) annotated genes in MSM 1, MSM 2, and MSM 3, respectively. Meanwhile, there are only 10.96% (25 genes), 10.23% (9 genes), and 12.32% (25 genes) annotated genes with integrity score over 50% in MSM 1, MSM 2, and MSM 3, respectively (Fig 5D and line 271-276 in the revised manuscript). Besides, among the isolated 42 unique gene fragments, ten gene fragments were identified to have a conserved coding sequence compared to their homolog in A chromosomes of *C. gibelio* or other species (S9 Table and line 318-320 and 653-657 in the revised manuscript).

It is also unclear for me how you designed primers to be specific or not of the MSMs fragments. This is important to evaluate if this is really a specific expression of the MSM genes.

**Response:** Thank you for your comments. PCR primers were designed following the subsequent rules. If the MSM fragment had homolog sequences (identity > 75%) in the reference genome without MSM, we designed the PCR primers according to the MSM fragment and made the 3’ end nucleotide of each primer locate at the different sites between MSM fragment and homologous sequence of reference genome. If the MSM fragment had no homolog sequence in the reference genome without MSM, we randomly designed the PCR primers according to the MSM fragment (line 669-675 in the revised manuscript).

The question of what do you called “clean reads” (see below) may be also important to understand and to evaluate what you have done on the assembly of MSMs.

**Response:** Thanks for your comments. We have provided the definition of clean reads, which refers to the sequencing reads after filtering low quality reads and removing adaptor sequences (line 212-213 in the revised manuscript).

More generally I think that your results are interesting and should deserve publication, but I would suggest again not to oversell your results with straightforward conclusions that are not well supported by your results. Being more cautious with conclusions will not decrease the quality of your results. I already mentioned that important point in my first review but there are still too many statements that are not well-supported by your results

**Response:** Thanks for your valuable suggestions, and we have toned down these statements of conclusions without solid evidence.

Specific comments:

Line 31-32: “Here, we identify a massively expanded satellite DNA cluster on microchromosomes of gibel carp via all-to-all similarity comparison”. Alone without further explanation this sentence cannot be understood by readers who will only read your abstract. An all-to-all similarity comparison does not mean anything if not described with more details

**Response:** Thanks for your comments. We have changed this sentence in the abstract (line 31-33 in the revised manuscript).

Lines 57-58: “Our previous studies have revealed that the supernumerary microchromosomes play a male determination role in gibel carp” I do not think that you can say that. Your previous studies revealed a correlation between some supernumerary microchromosomes and the male phenotype which could suggest that they MAY play a role in male determination. Same comment throughout your manuscript like for example lines 123-125 ‘ effects on sex determination [31-34]. In hexaploid C. gibelio, supernumerary microchromosomes in males have also been found to play a male determination role [23, 35]”

**Response:** Thank you for your comments and suggestions. We have checked through the whole manuscript and changed all the unprecise descriptions (line 28, 57 and 123 in the revised manuscript).

Lines 62-66: “These findings indicate that the male-specific microchromosomes resembling common features of sex chromosomes may be the main driving forces for male occurrence in gynogenetic gibel carp, through which the gynogenetic gibel carp can conquer the disadvantages of unisexual reproduction with evolutionary long existence”.

The first part of the sentence is OK except that it is not explained clearly (first mention here) what are the resemblance with sex chromosomes. For the second part this is pure speculation (and this sentence is also present in the abstract, the end of the introduction, the discussion …) and should not be mentioned without a lot more caution.

**Response:** Thanks for your advices. We have added the resemblance with sex chromosomes in the author summary (line 63-65 in the revised manuscript) and deleted the speculative sentences in the abstract, the introduction, and the discussion.

Line 136: what do you mean exactly by “sex related gene accumulation » ?

**Response:** We have changed “sex related gene accumulation” into “accumulation of genes with sex-specific or sex-biased expression” (line 135-136 in the revised manuscript).

Line 137: you did not provide any indication of a “and male determination function ». You could probably say that some genes on these B have a sex-specific or sex-biased expression profile and that’s all.

**Response:** Thank you for pointing out this statement that are not well-supported and we have deleted these words.

Lines 220-221: “All the contigs and corrected reads displayed much lower alignment level to the references in sharp contrast with the clean reads” what are clean reads? Isn’t it expected (or at least a possibility) to have B microchromosome reads remapping with a lower quality on a reference that do not have any B inside. If some B sequences have substantially evolved from their type A chromosome origin by only considering “clean reads” (if I am right in my own definition of your “clean reads”) you potentially removed a large part (potentially B-specific) of the B sequence.

**Response:** Thanks for your comments. We have provided the definition of clean reads, which refers to the sequencing reads after filtering low quality reads and removing adaptor sequences (line 212-213 in the revised manuscript).

Lines 229-231: “The total mapping rates to the reference genome of MSM 1, MSM 2, and MSM 3 were 0.46%, 0.13%, and 0.56%, respectively.” Does that mean that 1) only 0.46% pf MSM 1 are mapped on the reference genome OR 2) does it mean that 0.46% of the reference genome size is covered by MSM 1 reads? If 1) this is surprising given that you state that B are mainly made up from A regions. If 2) it needs to be better explained

**Response:** Thank you for pointing out this floppy mistake and we have changed this sentence. In fact, a total of 46.35% MSM 1, 38.84% MSM 2, and 40.01% MSM 3 were mapped to the reference genome, respectively (line 227-228 in the revised manuscript).

Lines 322-323: “trpv4 (transient receptor potential cation channel, subfamily V, member 4), which was suggested to be a possible regulator of sox9 [42, 43] and play a male differentiation role in the American alligator”. This is not totally correct to state that trpv4 plays a male differentiation role in alligator. Its expression is indeed induced by temperatures proximate to the TSD-related temperature in alligators and its chemical disruption also disturbed the expression pattern of gonadal transcriptomes. These are interesting correlations but do not really prove that it plays a role. Just a matter of being cautious in the wording again.

**Response:** Thanks for pointing out the incorrect wording. We have deleted these sentences as the trpv4 gene fragment does not contain a potential coding sequences.

Lines 359: what is the meaning of “variable” in “the evolution of variable sex chromosomes”?

**Response:** We have deleted the word “variable” to avoid ambiguity (line 359 in the revised manuscript).

Discussion: I found that the discussion on the evolutionary trajectory of the MSMs toward a future sex chromosome in a species that will ultimately have a transition from unisexual reproduction to sexual reproduction is pure speculation. Going back from now to the past is already not an easy exercise but projecting current results in the future is even more difficult. Personally, I would not discuss this.

**Response:** Thanks for your advice and we have removed the related discussions.

Lines 401-402: I am sorry but you cannot state that “Our present results strongly indicate that MSMs are responsible for male determination in C. gibelio [18, 23, 36]”. This is not to be nasty but you should refrain from overselling your nice results. Just keep conclusions that strictly fit with your results and it will still be an interesting paper. In that case you just provide additional evidence that MSMs could be … There is no strong indication as you mentioned only (and that’s already good) the characterization of MSM genes (or gene fragments) with a male-specific or male-biased expression.

**Response:** Thank you for your comments and suggestions. We have changed the exaggerated statements and rewritten this paragraph (line 391-393 in the revised manuscript).

I am not a native English speaker but could you double check that the following sentences are correct? Many sentences of this manuscript look weird to me and a detailed revision of English may be need.

**Response:** We have checked and revised the whole manuscript carefully and tried to avoid any grammatical errors.

“… is predicted to be extinction … “?

**Response:** We have changed this sentence to make it to be better understood (line 99 in the revised manuscript).

Line 122: “many species [30], which indicate supernumerary chromosomes may have functional” = indicate that?

**Response:** Thanks for your comment and we have deleted the second part of this sentence (line 122 in the revised manuscript).

Lines 389-390: « to be evolved supernumerary chromosomes … » to be evolved from?

**Response:** We have rewritten this sentence (line 385 in the revised manuscript).

**Reviewer #3:**

The authors improved the manuscript, but one question remained.

(Page 9, line 239 – 240) Considering that the sequence characterization of MSM1, MSM2 and MSM3 is based on the amplified DNA of single chromosomes, I strongly recommend toning down the statement “….We could deduce that the MSM 1 and MSM 3 had more similar sequence components, which were divergent from MSM 2.” I’m not aware of any WGA (including MDA-based) method which amplifies 1 to 1 the DNA of a microisolated chromosome. Very often, a bias is observed, which could easily explain the observed differences between MSM1, MSM2 and MSM3. Could it be in the end a single MSM chromosome type with many copies? Besides the DNA sequences of the miroisolated/amplified chromosomes, do you have any evidence (eg FISH data) that the MSMs differ in sequence composition from each other?

**Response:** Thanks for your valuable comments. We do not have additional evidence (eg FISH data) that the MSMs differ in sequence composition from each other, and there is a possibility that the MSMs may be copies of a single MSM chromosome type. Therefore, we have deleted these speculative statements.

Minor points:

Abstract, line 31 – 37

Please use past tense.

**Response:** Thanks for your suggestion. We have changed the present tense to the past tense in the abstract and the author summary (line 31-39 and 58-60 in the revised manuscript).

Page 5, line 117

Rephrase. “..accumulate organelle genomes”. Suggestion: “...accumulate organelle genome-derived sequences.”.

**Response:** We have revised this sentence according to your suggestion (line 117 in the revised manuscript).

Page 6, lines 154 – 160, Please rephrase, it is difficult to understand.

**Response:** Thanks for your comments. And we have revised these sentences (line 155-158 in the revised manuscript).

Page 7, line 187 and 17, line 454, Did you amplify the chromosomes individually?

If yes, rewrite like: ” ..and individually amplified microisolated chromosomes…”.

**Response:** Thanks for your suggestion and we have rephrased these sentences (line 186 and 445 in the revised manuscript).

Page 34, line 1037

Do you mean: Mitotic cells of three males were used as replication for co-localization analysis?

**Response:** Thanks for your question. We used mitotic cells of three males for co-localization analysis. And we have rewritten this sentence in the figure legend (line 454 and 1028 in the revised manuscript).

With thanks for your consideration.

Kind regards,

Sincerely yours,

Xi-Yin Li, Ph.D

Institute of Hydrobiology

Chinese Academy of Sciences

Wuhan 430072, China

Tel: +86-27-68780707

Fax: +86-27-68780123

E-mail: lixiyin@ihb.ac.cn
